# Genome sequencing and resequencing identified three horizontal gene transfers and uncovered the genetic mechanism on the intraspecies adaptive evolution of *Gastrodia elata* Blume

**DOI:** 10.3389/fpls.2022.1035157

**Published:** 2023-01-04

**Authors:** Yunsheng Wang, Muhammad Qasim Shahid

**Affiliations:** ^1^ School of Health and Life Science, Kaili University, Kaili, Guizhou, China; ^2^ State Key Laboratory for Conservation and Utilization of Subtropical Agro-Bioresources, South China Agricultural University, Guangzhou, China; ^3^ Guangdong Provincial Key Laboratory of Plant Molecular Breeding, South China Agricultural University, Guangzhou, China; ^4^ College of Agriculture, South China Agricultural University, Guangzhou, Guangdong, China

**Keywords:** adaptation, *Gastrodia elata*, horizontal gene transfers, population genomics, selection effect

## Abstract

Horizontal gene transfer is a rare and useful genetic mechanism in higher plants. *Gastrodia elata* Blume (*GE*) (Orchidaceae), well known as traditional medicinal material in East Asia, adopts a heterotrophic lifestyle, thus being considered to be more prone to horizontal gene transfer (HGT). *GE* is a “polytypic species” that currently comprised of five recognized forms according to the plant morphology. *G. elata Blume forma elata* (*GEE*) and *G. elata Bl.f.glauca* (*GEG*) are two common forms that naturally grow in different habitats with difference in altitude and latitude. *G. elata Bl.f.viridis* (*GEV*) often occurs sporadically in cultivated populations of *GEE* and *GEG*. However, the genetic relationships and genetic mechanism underpinned the divergent ecological adaptations of *GEE* and *GEG* have not been revealed. Here, we assembled a chromosome-level draft genome of *GEE* with 1.04 Gb. Among predicted 17,895 protein coding genes, we identified three HGTs. Meanwhile, we resequenced 10 *GEE* accessions, nine *GEG* accessions, and 10 *GEV* accessions, and identified two independent genetic lineages: *GEG*_pedigree (*GEG* individuals and *GEV* individuals collected from *GEG* populations) and *GEE*_pedigree (*GEE* individuals and *GEV* individuals collected from *GEE* populations), which strongly support the taxonomic status of *GEE* and *GEG* as subspecies, not as different forms. In highly differentiated genomic regions of *GEE*_pedigree and *GEG*_pedigree, three chalcone synthase-encoding genes and one Phox/Bem1p (PB1) domain of encoding Auxin (AUX)/Indoleacetic acid (IAA) were identified in selection sweeping genome regions, which suggested that differentiation between *GEE*_pedigree and *GEG*_pedigree was promoted by the selection of genes related to photoresponse and growth and development. Overall, this new genome would be helpful for breeding and utilization of *GE* and the new findings would deepen the understanding about ecological adaptation and evolution of *GE*.

## Introduction

Horizontal gene transfer (HGT), defined as the transfer of genetic material between non-mating species by certain means other than vertical inheritance from parents to offspring during reproduction, was firstly and frequently found to be a genetic process in prokaryotes such as viruses and bacteria (Griffith, 1928; Tatum and Lederberg, 1947). HGT plays an important role in species ecological adaption and evolution by endowing new and important traits, including antibiotic resistance, pathogenicity, photosynthetic capacity, and nitrogen fixation capacity, to the recipients (Ochman et al., 2000; Chen et al., 2021). HGT events have great implications for both basic and applied biological issues. For example, horizontally transferred genes show an inconsistent pedigree with that deduced from species phylogenies. It is also implied that individual genes or a group of genes generally cannot represent the whole genome for reconstructing the evolutionary relationship of species. Therefore, ‘Phylogenomics’ comes into being (Pascal et al., 2012), by which the tree of life has been reformulated (Burki et al., 2020). In recent years, accumulating evidence, especially that obtained from gene and genome sequencing studies, has shown that HGT is not limited to prokaryotes but can occur in a broad range of taxa. HGT is able to occur between prokaryotes and eukaryotes, and among cytoplasmic DNA, mitochondrial DNA, and nuclear genomes at different frequencies (Soucy et al., 2015). In addition, a large number of artificial gene transfers have been conducted in medicine and agriculture, which play an important role in ensuring food security and human health. The successful application of such artificial gene transfers is at least partly inspired by natural HGT. In eukaryotes, most HGT cases are found in protozoa, fungi, and animals (Slot and Rokas, 2011). In plants, most HGTs are from nuclear genome or plastid DNA to mitochondrial genome, with the rarest HGT cases observed with the plastid DNA as a recipient (Richardson and Palmer, 2007). Some HGT cases with higher plant nuclear genome as the recipient have been detected, in which the donor could be bacteria (Sieber et al., 2017; Husnik and McCutcheon, 2018), fungi (Shinozuka et al., 2020; Wang et al., 2020), or other kind of higher plants (Quispe-Huamanquispe et al., 2017; Dunning et al., 2019).

*GE* is a long-known traditional Chinese medicine that used to treat neurological and cerebrovascular diseases (Xu, 1992). Modern medical research has shown that *GE* has extensive health effects, including antidepressant, neuroprotective, anti-inflammatory, anti-dote, anxiolytic, and cognition-enhancing effects (Zhan et al., 2016). *GE* has no leaf nor root, and the tuber growing in the soil is the only vegetative organ. *GE* depends on its symbiotic fungi (i.e., *Mycena osmundicola* and *Armillaria mellea*) to provide organic nutrients for seed germination and tuber growth (Park and Lee, 2013). HGTs are frequently detected in parasitic and heterotrophic higher plant species that often closely contact at the tissue of epidermis or cortex with other species in the life circle (Xi et al., 2013; Molina et al., 2014; Yang et al., 2016). As a typical heterotrophic plant, it is not clear whether the HGT event had occurred in the *G. elata* genome.

*GE* naturally grows in mountainous areas of the narrow belt from northeast to southwest Asia, covering China, Siberia (in Russia), Northern Korea, Japan, Nepal, Bhutan, and India. According to the current taxonomy viewpoint proposed by Zhou et al. (1987), *GE* is a “polytypic species,” containing five different forms with different inflorescence colors and mature tuber shapes. *G. elata* Blume *forma alba* and *G. elata* Bl. *f. flavida* are the only forms found in the wild habitats with scarcity. *G. elata.* Bl. *f. elata* (*GEE*) and *G.elata* Bl*. f. glauca* (*GEG*) are commonly used as medicinal materials and are cultivated widely. *G. elata* Bl. *f. viridis* (*GEV*) often appears sporadically in cultivated populations of *GEE* and *GEG*. The inflorescence colors of *GEE*, *GEG*, and *GEV* are light red, reddish brown, and green, respectively. In general, the mature tubers of *GEE* are relatively slender, whereas those of *GEG* tend to be ellipsoidal. The mature tubers of *GEV* that scattered into *GEE* population are similar to that of *GEE*, and that scattered in *GEG* populations are similar to *GEG*. In addition to their significant morphological divergence, natural populations of *GEE* and *GEG* also occupy distinct niches with clear heterogeneous environments; the former are naturally distributed at altitudes in subtropical mountainous areas, while the latter are distributed in colder regions of subtropical mountainous areas, or in high latitude regions including Northeastern China, the Korean Peninsula, and the Russian Far East (Wang and Yu, 1999). However, the genetic relationships between different forms of *GE* remain unclear, and the genetic mechanism underlie the divergent ecological adaptability of *GEE* (800-1500 m altitude) and *GEG* (>1500 m altitude) remains unknown.

Here, we sequenced, assembled a chromosome-level draft genome of *G. elata* BI.*f.elata*, and resequenced 10 *GEE* accessions, nine *GEG* accessions, and 10 *GEV* accessions. The major aims of this study were to (1) contribute additional reference genome information for breeding and utilization of *GE*; (2) verify whether *GE* had experienced HGT events; (3) clarify the genetic relationships between *GEE*, *GEG* and *GEV*; (4) explore the genetic mechanism underlie the divergent ecological adaptability of *GEE* and *GEG.*

## Results

### Construction of a chromosome-level, high-quality genome assembly

We first performed a genome survey on a *G. elata* Bl.*f.elata* individuals (hereafter referred as G03) (See **Table S1** for more sampling information). K-mer analysis was executed based on ~ 53.54 Gb short reads that generated by the MGISEQ-2000 platform (MGI Tech Co., Ltd., Beijing, China), which revealed that G03 genome is about 1.09 Gb in size with a heterozygous rate of 0.33% (**Table 1**, **Tables S2, S3**; **Figure S1**). These results suggest that the genome size of *G. elata* BI.*f.elata* is smaller than that of *G. elata* Bl.*f.glauca* (1.18 Gb) (Yuan et al., 2018). We then generated approximately 130.50 Gb sub-reads with an average length of 17,007 bp using the PacBio Sequel II platform (Pacific Biosciences, Menlo Park, CA, USA) (**Table S4**). Based on these long-read sequencing data, we constructed a draft sequence assembly of about 1.04 Gb, with 367 contigs, an N50 of 16.87 Mb, and a GC contents of 34.29% (**Tables 1**, **S5, S6**). The size of this draft assembly covered 95.5% of the estimated genome size. We mapped short reads to this draft assembly to assess its quality, and more than 99.86% of the draft assembly were covered by short reads with > 20x coverage and only 0.04% InDel/SNPs were observed (**Tables S7, S8**), indicating that sequencing data is of high-quality and the draft genome assembly has high coverage. However, the Benchmarking Universal Single-Copy Orthologs (BUSCO) (https://busco.ezlab.org/) analysis showed that only 1,226 plant-specific orthologs (75.96% of the total 1,614 plant-specific orthologs) could be located in this assembly (**Table S9**), which was in contrasting to a with the above result that 95.5% of the estimated genome were assembled. This phenomenon may be explained by the unique characteristics of *G. elata*, such as the rootless and leafless morphology, and to obtain organic nutrients from its symbiotic fungi. These characteristics may incur gene loss in the evolutionary process of *G. elata* since it is needless to undertake corresponding metabolic functions as common plants do, which have been revealed and explained clearly by Yuan et al. (2018) and Xu et al. (2021). We obtained 379,187,951 paired-end Hi-C reads from Illumina sequencing, with 27.6% (201,724,768) mapped onto different contigs (**Table S10**). These Hi-C reads were applied to further anchor the contigs onto super-scaffolds or chromosomes. Finally, 198 contigs with a total length of 1.02 Gb were anchored onto the 18 chromosomes of *G. elata* (**Table 1**, **Table S11; Figure S2**), accounting for 98.65% and 94.20% of the contig-level assembly size and estimated genome size, respectively.

**Table 1:** Characteristics of G03 genome assembly.

Items	Counts
Estimated length	1.09 Gb
Total contig length	1.04 Gb
N50 of contig	16.87 Mb
Repetitive sequences ratio	77.94%
Number of predicted protein-coding genes	17,895
Total super-scaffolds length	1.02 Gb

### The annotated genes in the *G. elata* Bl.*f.elata* genome represent the smallest gene set among current genome sequenced angiosperms

**Figure 1:**
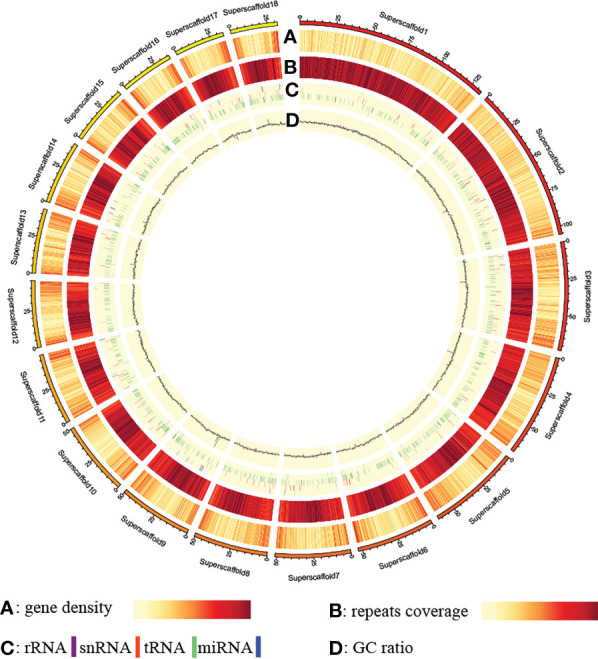
Distribution pattern of annotated elements along the genome.

Repetitive sequences, especially transposable elements (TEs), represent significant fractions of eukaryotic genomes and play important roles in gene regulation, chromosome structural organization, and genome evolutionary dynamics (Day et al., 2010). In contig assembly of G03 genome, about 808.2 Mb (77.94%) are repetitive sequences, of which 802.8 Mb are TEs dominated by long terminal repeats (648.6 Mb) (**Tables S12, S13**). A total of 17,895 protein-coding genes with an average length of 18,383.99 bp were identified in the G03 genome by a combination of three different methods, the average coding sequences and intron sequences of these protein-coding genes are 1,119.87 bp and 17,264.12 bp, respectively (**Table S14**). The number of annotated protein-coding genes of *G. elata* Bl.*f.elata* (17,895) is even smaller than that of *G. elata* Bl.*f.glauca* (18,969) reported by Yuan et al. (2018). Besides, non-coding RNA genes, including 25 miRNA, 1,098 tRNA, 204 rRNA, and 189 snRNA genes, were identified (**Table S15**; **Dataset 1**). Of the 17,895 predicted protein-coding genes, 15,029 (83.98%) could be functionally annotated, with 15,009 (83.87%), 11,270 (62.98%), 14,803 (82.72%), and 8,888 (49.67%) annotated by the NR, Swiss-Prot, KEGG, and GO databases, respectively (**Table S16**; **Dataset 2**). Chromosomal distribution analysis showed that the above-annotated elements were all unevenly distributed among the chromosomes of *G. elata* Bl.*f.elata* (**Figure 1**).

### Three HGT genes were detected in the *G. elata* genome

**Figure 2:**
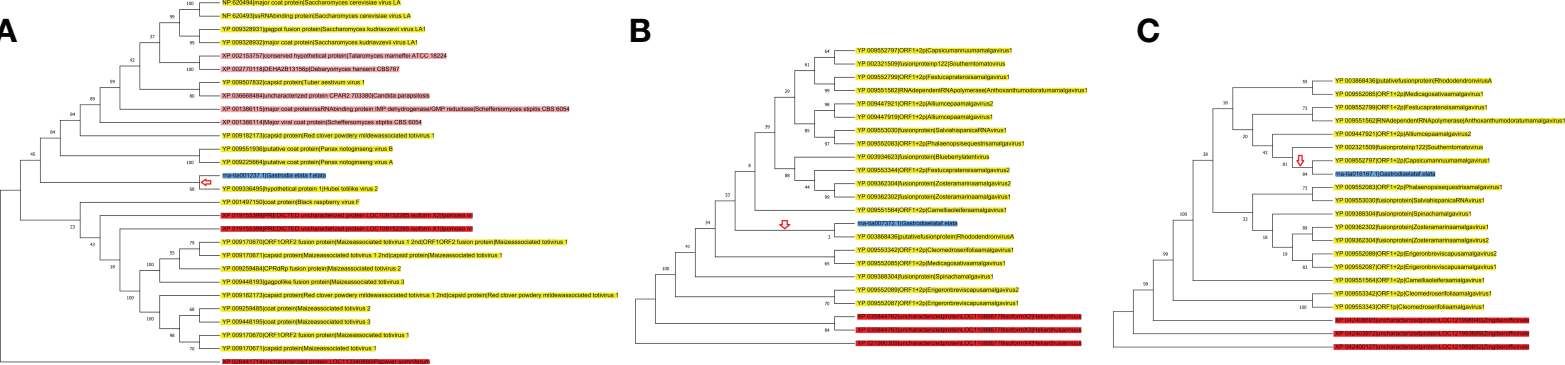
Maximum Likelihood (ML) phylogenetic tree of HGT homologs. **(A)** rna-tia001237.1; **(B)** rna-tia007372.1; **(C)** rna-tia016167.1. Terminal nodes highlighting with yellow, red, pink and blue backgrounds indicate viral, plant, fungi, and GE genes, respectively. Red arrow indicates HGTs, and numbers represent bootstrap values.

By using the methods suggested by Li et al. (2018), rna-tia001237.1 located on superscaffold1 (124604550^th^–124606754^th^ nucleotides), rna-tia016167.1 located on superscaffold8 (23043462^th^–23044569^th^ nucleotides) and rna-tia007372.1 located on superscaffold17 (25965883^th^–25966860^th^ nucleotides), were identified as HGTs by phylogeny and taxonomy distribution of annotated protein coding genes of G03. Phylogenetic trees of HGTs were constructed using homologs, which showed that the HTGs of *GE* were all from the virus genes (**Figure 2**; **Figures S3-S5**). Meanwhile, taxonomic distribution pattern of HTG homologs also showed that HTG homologs sequences in virus have the highest homology (based on the e-value of blast) with HTG sequences of *Gastrodia elata* (Dataset 3). Obviously, these three HTGs were evolved from viral genes. According to the all homologous trees, the HGT of rna-tia001237.1 happen between the virus donor to GE directly (**Figure S3**). However, HGT of rna-tia007372.1 and rna-tia016167.1 happened between the virus donor with the ancestor which shared by GE and other plant (**Figures S4, S5**). The GC contents of three HGTs were ranged from 49.6% to 56.8%, which were much higher than that of genome average value (34.29%) (**Table 2**). These results are consistent with the GC contents of genes in prokaryotes, which also usually high. The first HGT gene (rna-tia001237.1) harbored a 2,205-bp coding sequence without an intron and showed a low expression level in both tissues of G01 (*GEV*), and this gene was annotated as ‘uncharacterized protein’ by the Nr database and ‘major coat protein in the L-A virus’ by the InterPro database, which has high similarity to the gag-pol-like fusion protein in maize-associated totivirus 3 (**Dataset 4**). The second HGT gene (rna-tia016167.1) was composed of two exons with 1020 bp, and this gene expressed only in G02 (*GEG*), but higher expressions were detected in tuber than in flower. This gene was annotated as ‘protein FAM136A-like’ by the NR database and ‘Protein of unknown function DUF842, eukaryotic’ by the InterPro database (**Dataset 4**). The third HGT gene (rna-tia007372.1) was composed of three exons with 792 bp, which expressed in flower tissue of G01, G02 G03 and G04, and tuber tissue of G01, with the highest expression in flower of G04 (Dataset 4). The rna-tia007372.1 was annotated as probable monogalactosyldiacylglycerol synthase 3, chloroplastic isoform X1’ by the NR database (**Dataset 4**). Above results showed that rna-tia001237.1 is a young HGT and retained characteristics of the prokaryotic gene and has not been completely domesticated by the new host, while rna-tia007372.1 seem to be old HGTs and adopted by *GE*.

**Table 2:** Information of identified HGT genes.

Items	Counts		
	rna-tia001237.1	rna-tia016167.1	rna-tia007372.1
Location	1	8	17
Start position	124604550	23043462	25965883
End position	124606754	23044569	25966860
Gene length	2205	1108	984
Coding sequences length	2205	1020	792
Exon number	1	2	3
Predicted protein length	735	340	264
GC content (%)	56.8	49.8	49.6

Note: Terminal nodes highlighting with yellow, red, pink and blue backgrounds indicate viral, plant, fungi, and *GE* genes, respectively. Red arrow indicates HGTs, and numbers represent bootstrap values.

### Mapping of resequencing data to reveal genome coverage and distribution, and identification of single nucleotide polymorphisms (SNPs)

In total, 57,268,476-108,451,008 pairs of clean short reads composed of 8.5-16.2 Gb of clean data were obtained from the resequencing of 29 *GE* accessions, and more than 97.0% of reads were high quality (> Q20) in all samples, and 77.98-99.93% could be mapped onto the reference genome of G03 (**Table S17**). The average mapping depth was 6.51-12.78X and the mapping coverage percentage with at least 4X mapping depth reached to 74.67-96.44% for the whole genome sequence (**Table S18**). These results showed that the resequencing data had high genome coverage. We identified 1,524,081 high quality SNPs in *GE* population by analyzing the resequencing data.

### Phylogenetic and genetic structure analysis of subspecies taxonomic status of *GEE* and *GEG*

**Figure 3:**
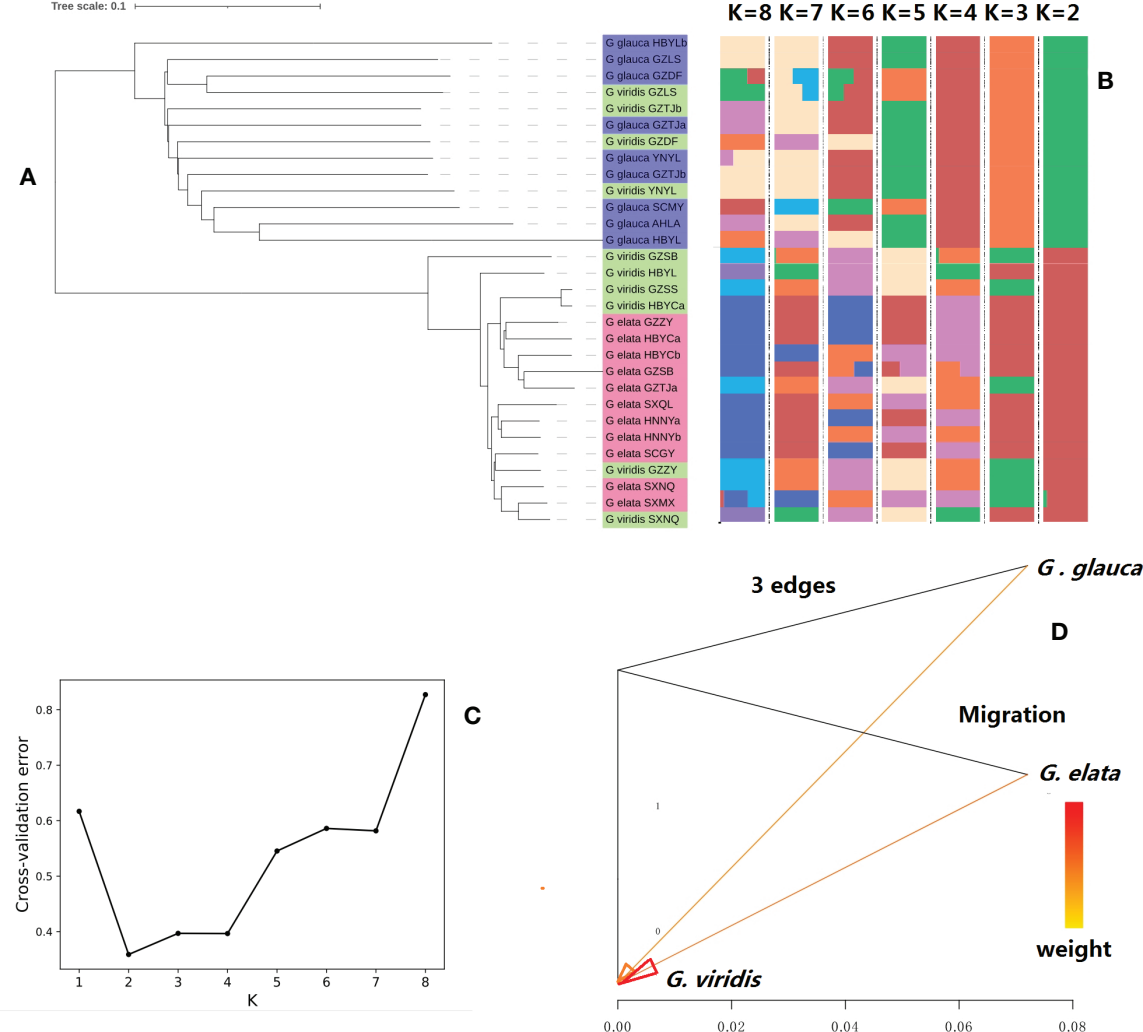
Population structure and gene flow analysis of 30 accessions of *Gastrodia elata (GE)* samples: **(A)** neighbor-joining phylogenetic tree; **(B)** admixture structure; **(C)**: the genetic population number (K) and its corresponding value of cross-validation error generated by admixture analysis (note: K = 2 denotes the lowest cross-validation error, suggesting that the samples should be clustered into two different genetic populations); **(D)**: gene flow among (G) *elata.f.glauca*, (*G*) *elata.f.elata*, and (G) *elata.f.viridis* populations. Note: Abscissa shows the value of gene flow and arrow indicates the direction of gene flow.

To exploit the genetic relationship of the collected *GE* samples, phylogenetic trees were constructed based on the nuclear genome data of 30 samples. Admixture structure analysis of all samples, based on the SNP data of the total samples, revealed that all samples clustered into two clear genetic lineages (**Figures 3A–C**). One was *GEE*_pedigree, which was composed of all *GEE* samples and *GEV* samples that grow within cultivated *GEE* populations, and other was *GEG*_pedigree that composed of all *GEG* samples and *GEV* samples that grow within cultivated *GEG* populations. Meanwhile, we constructed the phylogenetic tree of all samples by using their chloroplast and mitochondrial genome sequences separately (**Figure S6**), and their results were consistent with the pattern of nuclear genome tree i.e., *GEE*_pedigree and *GEG*_pedigree clustered at different ends. Based on the above results and the fact that *GEE* and *GEG* exhibit clear differences in plant morphology and eco-geographic distribution, we proposed that the taxonomic status of both *GEE* and *GEG* should be refined as subspecies, however, *GEV* is still classified as a mutant form, which may origin from both *GEE* and *GEG*. Our further analysis showed significant gene flow from *GEE* and *GEG* to *GEV* (**Figure 3D**), which strengthen our assumptions.

## Comparison of genetic structure of *GEE_pedigree* and *GEG_pedigree*

A total of 1,232,605, and 409,780 highly reliable single nucleotide polymorphisms (SNPs) were identified in the genomes of the *GEG*_pedigree and *GEE*_pedigree, and genetic diversities, as indicated by π values estimated by the nucleotide polymorphisms of pair differences, were 4.1919×10^−4^ and 0.8823×10^−4^, respectively (**Table 3**). However, if we classify all these samples into two populations, *GEV* and *non-GEV*, 2.915×10^−4^ and 3.175×10^−4^ genetic diversities were obtained, which is almost the same level of genetic diversity, respectively (**Table S19**). The π values of *GEG*_pedigree and *GEE*_pedigree were much lower than that of *Populus deltoides* (1.7×10^−3^) (Fahrenkrog et al., 2017), *Eriobotrya japonica* (1.0×10^−3^) (Wang and Paterson, 2021), *Malus domestica* (2.2×10^−3^) (Duan et al., 2017), *Manihot esculenta* (2.6×10^−3^) (Kawuki et al., 2009), *Vitis Vinifera* (5.1×10^−3^) (This et al., 2011), suggesting that *GEE*_pedigree and *GEE*_pedigree, especially *GEE*_pedigree, have a very low genetic diversity. This implies that there is a very limited scope for the genetic improvement of *GE.* The Tajima’s D values of *GEG*_pedigree, and *GEE*_pedigree populations at whole genome level were 1.3757, and −0.2211, respectively (**Table 3**). These findings tentatively indicated that divergent selection effects driven *GEG*_pedigree and *GEE*_pedigree to separate from each other.

**Table 3:** Genetic diversity and genetic differentiation of different populations.

Parameters	Population name		
	*GEG*_pedigree	*GEE*_pedigree	Total
Pi	4.1919×10 ^−4^	0.8823×10 ^−4^	3.0757×10 ^−4^
Tajima’s D	1.3757	-0.2211	-0.0498
SNP number	1,232,605	409,780	1,524,081
*F*_ST_	0.2513		
Dxy	0.2752		

GEG_pedigree: population including nine accessions of GEG and four accessions of GEV that grow in cultivated GEG populations; GEE_pedigree: population including ten accessions of GEE and five accessions of GEV that grow in cultivated GEE populations.

## Estimation of genetic differentiation of *GEG*_pedigree and *GEE*_pedigree

The average genetic differentiation index (Wright’s fixation index; *F*_ST_) and absolute genetic differentiation index (dXY) between the genetic populations of *GEE*_pedigree and *GEG*_pedigree were 0.2513 and 0.2752, respectively (**Table 3**). These values were higher than those of cultivated maize and its wild progenitor species (*F*_ST_ = 0.11) (Hufford et al., 2012), allotetraploid *Brassica napus* (AACC) and its one progenitor diploid species *B. rapa* (AA) (*F*_ST_ = 0.136), *B. napus* and another progenitor diploid species *B. oleracea* (CC) (*F*_ST_ = 0.246) (Lu et al., 2019). However, the *F*_ST_ between the genetic populations of *GEV* and non-*GEV* was only 0.0033 (**Table S19**). This analysis indicated a strong genetic differentiation between *GEG*_pedigree and *GEE*_pedigree populations, but almost no genetic differentiation between *GEV*_pedigree and non-*GEV*_pedigree. These results further prove the reliability of the abovementioned conclusions regarding the taxonomic statuses of *GEG*, *GEE*, and *GEV*.

### The selection sweeps and candidate genes responsible for local adaptions of *GEE_pedigree* and *GEG_pedigree*

**Figure 4:**
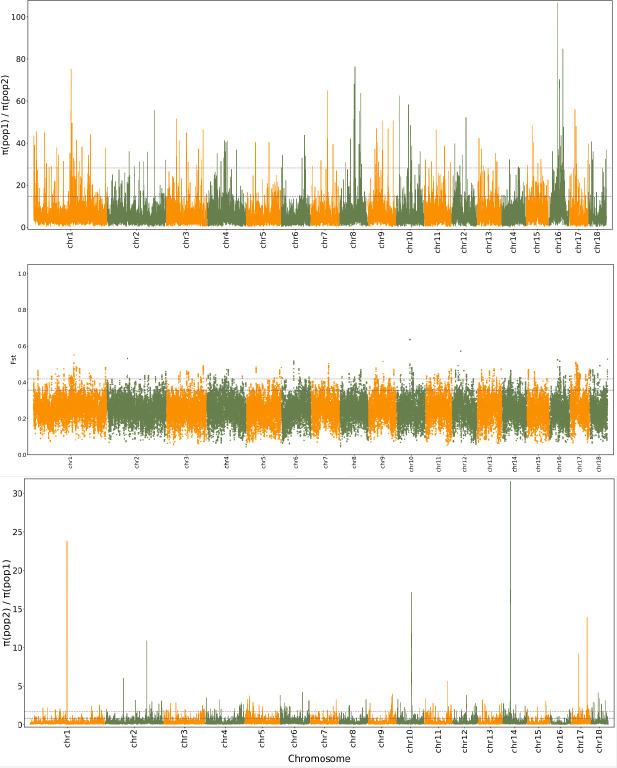
Distribution pattern of *F*_ST_ and π ratio along the chromosome between *GEE*_pedigree and *GEG*_pedigree. pop1 indicates GEG_pedigree (G. elata Bl. f. glauca [GEG] individuals and G. elata Bl. f. viridis [GEV] individuals collected from GEG populations) and pop2 indicates GEE_pedigree (G. elata. Blume forma elata [GEE] individuals and GEV individuals collected from GEE populations).

Note: pop1 indicates *GEG*_pedigree (*G. elata* Bl*. f. glauca* [*GEG*] individuals and *G. elata* Bl. *f. viridis* [*GEV*] individuals collected from *GEG* populations) and pop2 indicates *GEE*_pedigree (*G. elata.* Blume *forma elata* [*GEE*] individuals and *GEV* individuals collected from *GEE* populations).

Genomic regions with selective sweeps can be considered to be population-specific signatures of selection (Hoban et al., 2016). The ratio of the π values of *GEE*_pedigree and *GEG*_pedigree was used to identify the genomic regions that underwent selective sweeps, resulting in 256 (*p* ≤ 0.01 or top1) and 432 (*p* ≤ 0.05 or top5) genomic bins, holding 430 and 740 genes, respectively (**Figure 4**; **Table S19**; **Dataset 5**). However, for *GEG*_pedigree, there were 98 (*p ≤*0.01 or top1) and 173 (*p* ≤ 0.05 or top5) selective sweeping genomic bins, holding 240 and 442 genes, respectively (**Figure 4**; **Table S20**; **Dataset 5**). When these bins were further filtered by *F*_ST_ outliers, there were 79 (*p* ≤ 0.01 or top1) and 219 (*p* ≤ 0.05 or top5) selective sweeping genomic bins remaining in the whole genome of *GEE*_pedigree. In comparison, there were only 5 (*p* ≤ 0.01 or top1) and 17 (*p* ≤ 0.05 or top5) bins in the whole genome of *GEG*_pedigree (**Table S20**; **Figure 4**; **Dataset 5**). In total, 588 genes were identified to locate on selection sweeping region by the combined *F*_ST_ and dXY (*p* ≤ 0.01 or top1). These genes were involved in 125 specific metabolic pathways related to “cellular processes,” “environmental information processing,” “genetic information processing,” “metabolism,” and “organismal systems” (Dataset 6). These results imply that multi-gene interactions responded to natural selection from local environments and drove the divergence of *GEE*_pedigree and *GEG*_pedigree lineages. From the above mentioned genes and based on their functional annotation, we identified tia000401, tia000402, tia000403, and tia016287 as key players. The genes tia000401, tia000402, and tia000403 are located on superscaffold1 (44860831^th^–44985639^th^ nucleotides), and they encode chalcone synthase [EC:2.3.1.74] (K00660; Dataset 2, 6). Chalcone synthase plays crucial role in the first step of the flavonoid biosynthesis pathway (**Figure S7A**), and flavonoids are the major photoprotectants in plants (Schroder, 1999). These genes (tia000401, tia000402, and tia000403) were also involved in the “circadian rhythm-plant” process (**Figure S7B**), which play key roles in photoperiod adaptation in a given environment (Webb, 2003). Based on the above mentioned results, natural selection was found to have acted on the tia000401, tia000402, and tia000403 genes. As a result, *GEG*_pedigree and *GEE*_pedigree developed improved adaptions to their respective different light and temperature environments, which promoted their divergence. Meanwhile, we found that tia000402 displayed the highest expression, and expression levels were higher in flower than that in tuber tissues (**Dataset 7**). The gene tia016287 was located on extremely selective sweeps (Superscaffold8; 36507213^th^–36507782^nd^ nucleotides), where the ratio of pop1/pop2 was > 40 and *F*_ST_ was > 0.45. The gene tia016287 encodes a Phox/Bem1p (PB1) domain of Auxin (AUX)/Indoleacetic acid (IAA), which is an auxin-responsive protein (K14484) and involved in the auxin-activated signaling pathway (Dataset 2, 6). Previous study showed that Auxin regulates growth and development of plants (Leyser, 2001). This implies that the strong selection effect on gene tia016287 probably promoted divergent adaption regarding the growth and development of *GEG*_pedigree and *GEE*_pedigree. So, tia000401, tia000402, tia000403 and tia016287 genes can be regarded as the key candidates of ‘speciation genes’ in *G. elata*.

## Discussion

In recent years, with the emergence of advanced sequencing technologies, HGT has re-attracted wide attention for its unique roles in phylogeny and adaptive evolution of species (Yoshida et al., 2010; Yang et al., 2019). Meanwhile, different and even opposite views on the HGT have also been proposed, including its frequency and evolutionary significance in higher plants (Baltrus, 2013; Huang, 2013). Some researchers argue that HGTs in eukaryotes are overestimated due to the contamination of bacteria or other microorganisms in the process from DNA/RNA extraction to sequencing, especially genomic data used for HGT detection are based on short reads generated by the next-generation sequencing platforms (Koutsovoulos et al., 2016; Richards and Monier, 2016; Wickell and Li, 2020). In this study, three HGT genes were identified from the reference genome of *G. elata* BI.*f.elata*, which was assembled based on long-read sequencing data, and were located on specific chromosomes. Meanwhile, mRNA sequences of all three HGT genes could be identified in at least one transcriptomic dataset generated from flower or tuber tissues of four different accessions of *GE* (Dataset 4). Moreover, we identified the highly homologous sequences of each HTGs from other three published draft genomes of *GE* (Dataset 4). Therefore, these observations eliminated the possibility that HGT genes detected in this study were the results of contamination. BLAST searches and gene tree–species tree reconciliation are two effective and common methods to detect HGTs (Wickell and Li, 2020), and both of them were conducted in this study, which confirmed that horizontal transfers of these three genes were true. However, the proportion of HGT genes (3 out of 17,895 protein-coding genes) in the *G. elata* genome is low. Considering the results of previous studies of HGTs in other higher plants (Richards et al., 2009; Koutsovoulos et al., 2016; Yang et al., 2019), it is possible to conclude that the HGT in higher plants is a rare genetic event. Previous studies suggested that HGTs are frequently detected in parasitic and heterotrophic higher plant species, because two different taxa contact their tissues directly (Davis and Wurdack, 2004; Zhang et al., 2014; Yang et al., 2019). However, we did not detect the footprint of HGTs from fungi to *GE* in this study by comparing with the annotation results of chloroplast and mitochondrial genes of *GE* by Yuan et al. (2018), who also detected no HGTs from fungi to cytoplasmic genome, which revealed that the direct contact may not be only requirement for HTG event. The three HTGs that we identified in the present study were all from viruses, which may happen when plant viruses rely on plant cells for reproduction, and they will turn on the reverse transcription mechanism and convert their genetic material into double stranded DNA, thus having the opportunity to insert their genetic material into the plant nuclear genome. This is in accordance with the laws of classical genetics.

Subspecies represent a lower unit of species (Haig et al., 2006). From an evolutionary point of view, they lie in a place of continuous variation between a population and a known species (John, 2010). This variation may represent adaptation to the heterogeneous local ecological environment (Winker, 2010), which will eventually lead to the emergence of new species. Thus, subspecies have long been regarded as “incipient species” (Rothschild and Jordan, 1895). In recent years, increasing attention has been focused on using subspecies as a tool to study the initial stage of speciation (Kopac et al., 2014; Schmidt et al., 2019; Marin et al., 2020). Traditionally, subspecies were defined as groups of individuals that could be discriminated according to their morphology and geographical distribution (Amadon, 1949). However, in some cases these definitions have since been found to be mismatched with phylogenetic clusters identified using modern molecular methods (Phillimore and Owens, 2006). Thus, the new concept of subspecies is defined as “genetically differentiated populations within a species that have unique morphology or demonstrated a difference in adaptation to the local environment” (Haig et al., 2006), or as “subset of populations with consistent genetic differences from other subsets of populations at multiple independent loci, with genetic differences consisting of significant variation in microsatellite alleles and mtDNA haplotype frequencies, the presence of unique alleles or haplotypes, and significant net sequence divergence” (Funk et al., 2007). This emphasizes the diagnosability of genealogy and ecological adaptation. *GEG* and *GEE* have differentiated to the point where they have different diagnosable botanical characteristics and different geographical distributions. Here, *GEG_*pedigree and *GEE_*pedigree were found to contain two independent genetic and evolutionary lineages. Therefore, based on either the old or new concepts of subspecies, *GEG* and *GEE* should be redefined as *G. elata* Bl*. ssp. glauca* and *G. elata* Bl. *ssp. elata*, respectively. Of course, *GEG* and *GEE* have high cross compatibility and hybrid fertility (Wang and Yu, 1999), determining that they are not separated species. Based on the above, we uncover the variation pattern of *GEE, GEG*, together with *GEV* at genome wide in this study, and corresponding results refined *GEE* and *GEG* as subspeices, which can deepen the understanding not only on the ecological adaption, but also on incipient speciation of *GE*.

Uncovering the molecular mechanisms of ecological adaption is a central target in evolutionary biology (Berlocher and Feder, 2002). Two ideologies, “forward-ecology” and “reverse-ecology,” were used for the above-mentioned target. The former involves identifying those traits that are significantly related to ecological adaption or reproductive isolation, and then investigating the genes underlie said traits. The latter involves identifying the gene loci or genomic regions that have undergone a selection effect, and then screening those genes whose molecular functions are related to ecological adaption and reproductive isolation (Li et al., 2008). In recent years, “reverse-ecology” has become more popular because of ease in high-throughput sequencing and resequencing technology (Ungerer et al., 2008; Levy. and Borenstein, 2012). Meanwhile, methods including *F*_ST_ outliers and comparing the nucleotide polymorphism ratios of a pair of lineages to identify the selection sweep genomic region or genes/alleles that was regarded as the most effective candidate of molecular basement responsible for the ecological adaption differentiation or speciation between two populations have been developed and widely applied (Axelsson et al., 2013; Ravinet et al., 2017; Jiao et al., 2020; Yang et al., 2022). Here, lots of selection sweeps located at different genome positions of *GEE_*pedigree and *GEG_*pedigree were found. Within these genome region, hundreds of genes were identified and executed a wide range of biological functions, suggesting that multi-gene interactions responded to the natural selection from native environments for local adaptation of *GEE_*pedigree and *GEG*_pedigree. However, some genes may have been more critical, playing leading roles, while others may have been in more passive, subordinate positions. Among selection sweeping genes, we ranked tia000401, tia000402, tia000403, and tia016287 as key genes according to their biological function, and can be the major candidates of ‘speciation gene’ of *G. elata. ssp. elata* and *G. elata. ssp. Glauca*, but this need further experiments to be verified.

## Conclusions

Here, we assembled and annotated a high-quality chromosome-level reference genome of *GE* (1.05 Gb), containing 77.94% repetitive sequences and 17,895 putative protein-coding genes including three HTGs. Moreover, ten *GEE* accessions, nine *GEG* accessions, and ten *GEV* accessions were resequenced and analyzed. The population genomic analysis conducted here strongly suggests that *GEE_*pedigree and *GEG_*pedigree should be regarded as subspecies, instead of as different forms (i.e., the current viewpoint). Meanwhile, three chalcone synthase encoding genes and one PB1 domain of the AUX/IAA encoding gene were found to have strong selection effects. Therefore, these genes were ranked as the most important candidates for *GEG_*pedigree and *GEE_*pedigree, which influence the adaptation of the divergent photoperiod and temperature and the regulation of inconsistent growth and development characteristics. In short, this study provides an abundance of genomic information for further research and utilization of *G. elata*, and proposed a new view on the classification status of *G. elata.f.elata* and *G.elata.f.glauca*, and deepen the understanding of adaptive evolution of *GE*.

## Materials and methods

### Plant materials

The tuber tissue of a two-year-old *G. elata* Bl.*f.elata* strain (G03) plant taken from Yiling county, Yichang city, Hubei province of China was used as sequencing material for draft genome assembly. The flower and tuber transcriptomes of same individual were used to assist in the predicting and annotating of protein-coding genes of the draft genome. We sampled the flower of nine accessions of *GEG*s from wild populations, 10 accessions of *GEE* from wild populations, and 10 accessions of *GEV* from cultivated populations to use for resequencing. The flower of *GEV* were offered by owner named as Rui Wang, Guangwen Zhang, Youmin Li, Xiaojun Chen, Xiaohong Liu, Heng Wang, Dali Huang, Jingwu Liu, Guisheng Tao and Juan Zhao. More detailed information on these samples is listed in **Table S1**. The specimens of the above materials were kept in the laboratory of Kaili University.

### DNA preparation, short-read library construction and sequencing

Total DNA of 30 *GE* accessions were extracted using the CTAB protocols proposed by (Doyle and Doyle, 1987). For each sample, the qualified DNAs were sheared into 300-500 bp fragments using Covaris ultrasonic breaker (Covaris, Woburn, MA, USA). Then the fragments were repaired by adding a tail and an adaptor sequence, and purified for sequencing library construction. The constructed library was sequenced on a MGISEQ 2000 platform (BGI, Shenzhen, China) to produce raw reads. The raw reads were then filtered by using the following criteria: filter out the adaptor sequences; remove duplicate reads produced by PCR amplification; remove the corresponding paired-end reads, if the N content exceeds 10% of the length of a single read; and remove the corresponding paired-end reads, if the low-quality base (≤ 5) number exceeds 50% of the length of a single read. The remaining clean reads with Q > 20 were used for further analyses.

### Long-read library construction and sequencing

Long-read library was constructed with the following steps. The qualified DNA extracted from G03 was sheared into ~ 30 kb fragments by Covaris g-TUBE (Covaris, Woburn, MA, USA). DNA fragments were enriched and purified by AMPure XP magnetic beads (Beckman Coulter Inc, Brea, California, America), and then were damage- and end-repaired. The SMRT dumbbell-type adapters were ligated at both ends of DNA fragments. DNA fragments without adapters were removed by exonuclease to obtain the initial sequencing library. The Bluepippin system (Sage Science, Beverly, MA, USA) was used to screen the initial sequencing library to obtain the final sequencing library. The quality of the final sequencing library was determined by a Qubit 2.0 fluorometer (Thermo Fisher Scientific, Waltham, MA, USA) and the library size was estimated using an Agilent 2100 bioanalyzer (Agilent Technologies, Palo Alto, CA, USA). The qualified library (OD260/280 = 1.8-2.0; concentration > 50 ng/μL) was sequenced on a PacBio RS II platform (Pacific Biosciences, Menlo Park, CA, USA). The SMRTlink software (https://www.pacb.com/support/software-downloads/ ) was used to filter and process the resulting data with ‘minLength = 500’; other parameters were used as default settings.

### RNA extraction, cDNA library construction and RNA sequencing

Total RNA was extracted from the flower and tuber tissues of strain G01, G02, G03, and G04 (Please see **Table S1** for samples detail) by strictly following the guidelines of the RNA extraction kit (Thermo Fisher Scientific Inc, Waltham, MA, USA). The concentration and purity of the extracted RNA were measured by a Nanodrop 2000 (Thermo Fisher Scientific, Waltham, MA, USA) spectrophotometer, while RNA integrity and RNA integrity number (RIN) were measured by agarose gel electrophoresis and an Agilent 2100 bioanalyzer (Agilent Technologies, Palo Alto, CA, USA), respectively. Approximately 2 μg of high-quality RNA showing a clear band in the agarose gel, a concentration ≥ 300 ng/μL, and OD_260_/OD_280_ = 1.8-2.2 was used to construct the sequencing library according to the recommended protocols by the manufacturer. mRNA was enriched from total RNA by using magnetic oligo(dT) beads. mRNA was sheared into fragments by Covaris g-TUBE (Covaris, Woburn, MA, USA). The first-strand cDNA was synthesized by the M-MuLV reverse transcriptase system using mRNA fragments as template and random oligonucleotides as primers. Then mRNA was degraded by RNaseH, and the second-strand cDNA was synthesized with dNTPs in the DNA polymerase I system. The purified double-stranded cDNA was end-repaired by adding an A-tail and ligated with the sequencing adaptor. The 250-300 bp cDNA fragments were screened out by AMPure XP beads and amplified by PCR. The PCR products were again purified by AMPure XP beads to obtain the final sequencing library. Finally, RNA sequencing was performed on an Illumina HiSeq 4000 platform (Illumina, San Diego, CA, USA) to generate more than 6 Gb raw data per sample.

The clean data were obtained by filtering out the reads containing the adaptor sequence, and paired-end reads containing N content exceeds 10% of the length proportion, or low-quality (< = 5) base number exceeds 50% of the length proportion in a single read. Only high-quality clean data were used for further analyses. The data outputs including the number of sequencing reads, sequencing error rate, Q20 content, Q30 content, and GC content were counted.

### RNA sequencing and gene expression analysis

The reference transcripts were generated from qualified clean RNA reads (> Q20) of the flower G01, G02, G03 and G04 through mapping onto the assembled reference genome of G03. Then clean reads were mapped onto the transcripts using Bowtie2 v2.3.4.1 (https://wiki.rc.usf.edu/index.php/Bowtie2#Version ). If a transcript sequence mapped onto the predicted protein-coding region of the reference genome, then it was considered as an existing gene, otherwise the corresponding sequence was considered as a new gene. The number of reads mapped on each transcript in each sample were counted based on the mapping results produced by Bowtie2. Subsequently, the FPKM (fragments per kilobase per million bases)-transformed results were generated using RSEM software (http://deweylab.github.io/RSEM/) and the expression patterns of protein-coding genes or their transcripts were obtained.

### Genome survey by K-mer analysis

The estimation of the G03 genome size (C-value) was performed based on short-read sequencing data using the KAT program (Mapleson et al., 2017) according to the following formula: genome size = (total nucleotide number)/(average sequencing depth) = (total number of K-mer)/(average K-mer coverage). The K value was used based on the maximum number of odd numbers that fit the following formula: 4^K/genome > 200.

### Construction of the contig-level draft genome assembly

The MECAT software (Xiao et al., 2017) was used to assemble an initial draft genome assembly of G03 based on long-read sequencing data, then the ARROW software built in the Smrtlink package (https://www.pacb.com/support/software-downloads/ ) was used to correct any errors in the draft genome assembly with a minimum coverage of 15, and the other parameters were kept at default settings. The corrected draft genome assembly was polished using Illumina short reads to obtain a final draft genome assembly by Pilon software (Walker et al., 2014).

### Hi-C library construction and sequencing

Tuber tissues of G03 were pretreated according to the following steps. The DNA conformation of the cells was first fixed with paraformaldehyde. The cells were lysed, and the cross-linked DNA was digested by restriction endonucleases, *Bal*I, *Eco*RI, and *Bam*HI, to produce sticky ends, which were then repaired and introduced to be labeled with oligonucleotide ends containing biotin. DNA fragments were ligated using DNA ligase, and the cross-linked DNA was removed by protease digestion and purification. DNA fragments were randomly divided into 300-500 bp groups, and the labeled DNA was captured by avidin magnetic beads for Hi-C sequencing library construction as follow. The biotin-containing DNA was captured through the adsorption of avidin magnetic beads. Then the DNA fragments were end-repaired by adding poly-A tailed and ligated adapter. The PCR amplification was performed, and the Hi-C library was purified by gel electrophoresis. A Qubit 2.0 fluorometer was then used for preliminary quantification of the library, and the library was diluted to a concentration of 1 ng/μL. Then, an Agilent 2100 bioanalyzer was used to determine the insert size of the library, and the effective concentration of the library was accurately quantified by qPCR. Finally, the qualified library was sequenced on an Illumina HiSeq platform (paired-end sequencing, 2 × 150 bp) according to the effective concentration and the demand of the target data.

### Hi-C scaffolding

Raw reads generated by the Illumina HiSeq platform (paired-end sequencing, 2 × 150 bp) were filtered out those containing the adaptor, continuous bases with a quality value < 20 at both ends, and those with a final length of < 50 bp in any single read. The BWA v0.7.17 software (https://sourceforge.net/projects/bio-bwa/files/ ) was used to map the clean reads of G03 draft genome assembly. The reads with a distance from the restriction site > 500 bp were removed, and the remaining data were used to construct a chromosome-level genome assembly by Lachesis (Burton et al., 2013). The interaction map was constructed for the assembled chromosome-level genome by Juicer, and the visual error correction was carried out by Juicebox (Durand et al., 2016).

### Evaluation of completeness and accuracy of the *G. elata* Bl.*f.elata* genome assembly

The Benchmarking Universal Single-Copy Ortholog (BUSCO) analysis was used to estimate the completeness of the *G. elata* Bl.*f.elata* genome assembly by searching for plant-specific orthologs against the embryophyta_odb9 dataset (https://busco.ezlab.org/ ). The possible loss rate was calculated based on the single-copy orthologous gene set in OrthoDB (http://cegg.unige.ch/orthodb6). The short reads were mapped to the *G. elata* Bl.*f.elata* genome assembly by BWA, and SNP calling and filtering were performed by GATK (https://www.broadinstitute.org/gatk/). Homozygous and heterozygous SNPs and InDels were calculated to assess the accuracy of the genome assembly.

### Annotation of the *G. elata* Bl.*f.elata* genome

First of all, repetitive sequences in the *G. elata* Bl.*f.elata* genome assembly were annotated based on the Repbase database (https://www.girinst.org/repbase/ ). Three methods were used, including (1) homology-based prediction using RepeatMasker v4.09 and RepeatProteinMask) (Smit et al, 2019); (2) self-sequence alignment-based prediction using RepeatModeler v1.0.11 (Smit and Hubley, 2017), RepeatScout v1.0.5 (Price et al., 2005), and the tandem repeats database (TRDB) (http://tandem.bu.edu/trf/trf.html); and (3) *de novo* prediction using LTR-FINDER (Xu and Wang, 2007).

Secondly, protein-coding genes were initially predicted by integrating multiple prediction methods, including homology-based prediction (based on at least two to three related species) (Keilwagen et al., 2016); *de novo* prediction using Augustus v3.3 (Stanke and Morgenstern, 2005), GENSCAN (Burge and Karlin, 1997), and GlimmerHMM software v3.0.4 (Majoros et al., 2004); and transcriptome-based prediction (Trapnell et al., 2010) assisted by clean RNA sequencing data from the flower and tuber tissues of G03. Then, the various gene sets obtained from different methods were integrated into a non-redundant and more complete gene set using MAKER v2.00 (Cantarel et al., 2008). Meanwhile, through the integration of BUSCO results, the final reliable gene set was obtained by using the HiCESAP pipeline developed by Maiwei Int. Finally, functional annotation of the protein-coding genes was carried out by searching against the frequently used protein databases including Swiss-Prot (https://www.expasy.org/resources/uniprotkb-swiss-prot), TrEMBL (http://www.bioinfo.pte.hu/more/TrEMBL.htm), KEGG (https://www.genome.jp/kegg/), InterPro (https://www.ebi.ac.uk/interpro/), GO (http://geneontology.org/), and NR (https://www.ncbi.nlm.nih.gov/refseq/about/nonredundantproteins/).

Thirdly, tRNAscan-SE software (Lowe and Eddy, 1997) was used to identify tRNA sequences in the G03 genome assembly according to the structural characteristics of tRNAs. BLASTN searches (https://blast.ncbi.nlm.nih.gov/Blast.cgi ) were conducted to identify rRNA sequences in the G03 genome using rRNA sequences in the related species as reference. miRNAs and snRNAs in the G03 genome were predicted using the covariance model and INFERNAL v1.1 (Nawrocki and Eddy, 2013) based on Rfam v14.1 (Griffiths-Jones et al., 2005).

Finally, software Circos (Krzywinski et al., 2009) was used to visualize component compositions on the chromosomes based on the above annotation results of repetitive sequences, protein-coding genes, and rRNA genes.

### HTG identified

We identified HTGs according to the methods explained by Li et al. (Li et al., 2018). In brief, all protein sequences of annotated genes of G03 were blastp against the Refseq (https://www.ncbi.nlm.nih.gov/refseq/) and Nr databases (https://www.ncbi.nlm.nih.gov/refseq/about/nonredundantproteins/) with p-values lower than 0.05, and classified hits of each query to define taxonomic group (including plant, archaea, bacteria, fungi, virus, other). Then extract best bit-score with e-values in top 20 and calculated the HGT index (h) (Boschetti et al., 2012) and alien index (AI) (Gladyshev. et al., 2008) for each remaining query, and deduce the viable HGT when a query by both h index and AI index led to similar conclusion to be possible HGT. Finally, HTGs were determined by the phylogenetic tree of candidate HTG homologs that were constructed by measuring the maximum likelihood using software TBtool v.1.1043 (https://github.com/CJ-Chen/TBtools/releases) under the GTR + g model with 5000 bootstrap (https://github.com/stephaneguindon/phyml), and taxonomic distribution of HTG homologs.

### Resequencing and clean data mapping

The draft genome sequence at the chromosome level of *GEE* (G03) was used as a reference genome for mapping the clean resequencing data using the BWA software (https://github.com/lh3/bwa). The Picard software (https://sourceforge.net/projects/picard/) was used to perform duplicate mapping. The SAMtools software (https://github.com/samtools/samtools) was used to determine the mapping rate, coverage, and sequencing depth.

### SNP calling and filtering

The GATK software (https://gatk.broadinstitute.org/hc/en-us ) with default parameters was used to call the SNPs. Those SNPs with supporting read numbers < 4, minimum allele frequency < 0.05, and sample coverage proportion >10% (sample coverage) are considered as highly reliable, and the remaining high-reliability SNP dataset with more than 50% sample coverage was used for further analysis. The genome coverage of resequencing data was more than 90% for all samples used for SNP identification in the present study (**Table S18**). The ANNOVAR software (https://www.openbioinformatics.org/annovar/annovar_download.html ) was used to annotate the SNPs detected in each sample, including location information and type of variation.

### Population structure analyses

Three analyses, including phylogenetic tree construction, principal component analysis (PCA), and genetic structure analysis, were performed based on the population SNP dataset. The phylogenetic tree was constructed using the neighbor-joining method (Saitou and Nei, 1987), based on a distance matrix calculated using the Treebest software (Vilella et al., 2009). Similar approach was used to construct phylogenetic tree based on chloroplasts and mitochondria sequences of all samples with kiwifruit (*Actinidia chinensis*) and citrus (*Citrus reticulata*) as outgroups. The PCA of samples was performed using the GCTA software (Yang et al., 2011). The population structure was analyzed using the Admixture software, based on a Bayesian mathematical model (Alexander et al., 2009). The purpose of this study was to classify each material into a specific group and determine the population structure of the total population. The best K value was determined by calculating the cross-validation error, and the minimum cross-validation error was used to determine the best K value. The gene flows among *GEE*, *GEG* and *GEV* was calculated by program treemix (version:1.1.3) (https://bitbucket.org/nygcresearch/treemix/downloads/ ).

### Divergence outlier identification, selection effect, and selective sweeping

Genomic differentiation between populations was measured by *F*_ST_ (Weir and Cockerham, 1984) and dXY (equation 10.20) (Nei, 1987). Genomic bins with the highest *F*_ST_ (p ≤ 0.01 or top1) and/or dXY (p ≤ 0.05 or top5) values were considered outliers, and GIS. The selection effect on populations was analyzed using Tajima’s D values (Tajima, 1989). The effect of selection sweep was estimated by calculating the ratio of π (θ) values (Schlötterer et al., 2004) and by a combined *F*_ST_-π approach (Akey et al., 2002). The former was used to define genomic regions where the nucleotide diversity of π_pop1_/π_pop2_ (or π_pop2_/π_pop1_) reduced sharply (p ≤ 0.01 or ≤ top1) as selective sweeps, whereas the latter was used to define genomic regions where the nucleotide diversity of π_pop1_/π_pop2_ (or π_pop2_/π_pop1_) decreased sharply (p ≤ 0.01 or ≤ top1) accompanied by a relatively high *F*_ST_ (p ≤ 0.01 or ≤ top1). The Tajima’s D values, *F*_ST_, dXY, and π (θ) were all computed using the VCFtools software (http://vcftools.sourceforge.net/)].

### Functional annotation and enrichment analysis of genes identified in selective sweeps

The genes located in genomic blocks that underwent section sweeps were further annotated for molecular or biological functions using the BLASTX algorithm (E value < 1.0 E^− 5^) by querying them against the following databases: Nr, TrEMBL, GO, COGs, and KEGG. The GOseq software (Young et al., 2010) was used for GO enrichment analysis and the KOBAS software (Mao et al., 2005) was used for KEGG enrichment analysis. According to aforementioned annotation, we focused on several genes in the sweeping region, which response to biological rhythm or development that adaptive for light and temperature environment.

## Data availability statement

The draft genome and predicted protein-coding gene of G03 with gff format, re-sequencing raw data of 29 GE accessions, and flower and leaf transcriptome raw data of G01, G02, G03 and G04 have been deposited in China National Center for Bioinformation (https://bigd.big.ac.cn/?lang=zh) (Accessible ID: GWHAOSR00000000; CRA005104; OMIX677, and OMIX002622).

## Ethics statement

Plant samples used in the study were not collected from national park or natural reserve. According to national and local legislation, no specific permission was required for collecting these plants. We confirm that this complies with national guidelines and no formal ethics approval was required in this particular case.

## Author contributions

YW designed the experiments, collected samples, analyzed data, YW and MS wrote and revised the paper. All authors contributed to the article and approved the submitted version.

## Supplementary material

Supplementary File 8 This file contains **Supplementary Figures 1-7**, **Supplementary Tables 1-20**.
