# Supplementary material for: Genome sequencing and resequencing identified three horizontal gene transfers and uncovered the genetic mechanism on the intraspecies adaptive evolution of Gastrodia elata Blume
Source: Front Plant Sci. 2023 Jan 4;13:1035157. doi: 10.3389/fpls.2022.1035157 (PMC9848658; doi:10.3389/fpls.2022.1035157)
Supplement: Supplementary File 8 — This file contains Supplementary Figures 1-7 , Supplementary Tables 1-20 . [file DataSheet_8.pdf]

Genome sequencing and resequencing identified three horizontal gene transfers and uncovered the genetic mechanism on the intraspecies adaptive evolution of *Gastrodia elata* Blume

Yunsheng Wang, Muhammad Qasim Shahid

Supplementary Figures and Tables

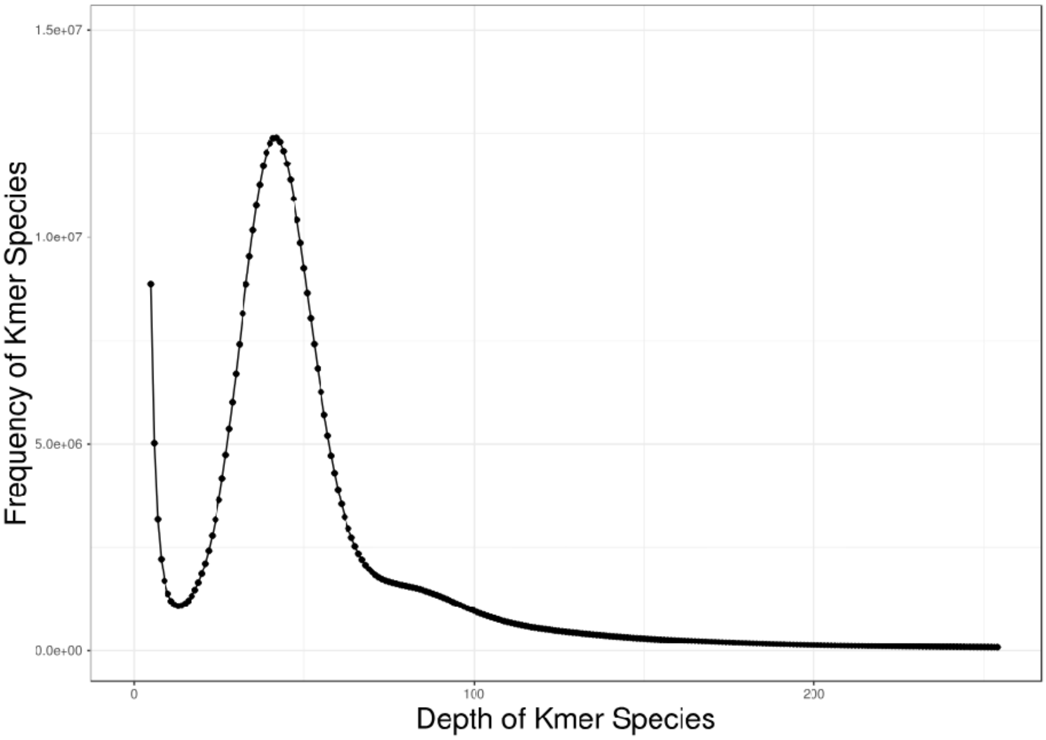

Figure S1 Genome survey based on the frequency distribution of k-mers (k = 17) of *GEE* genome-based short-reads

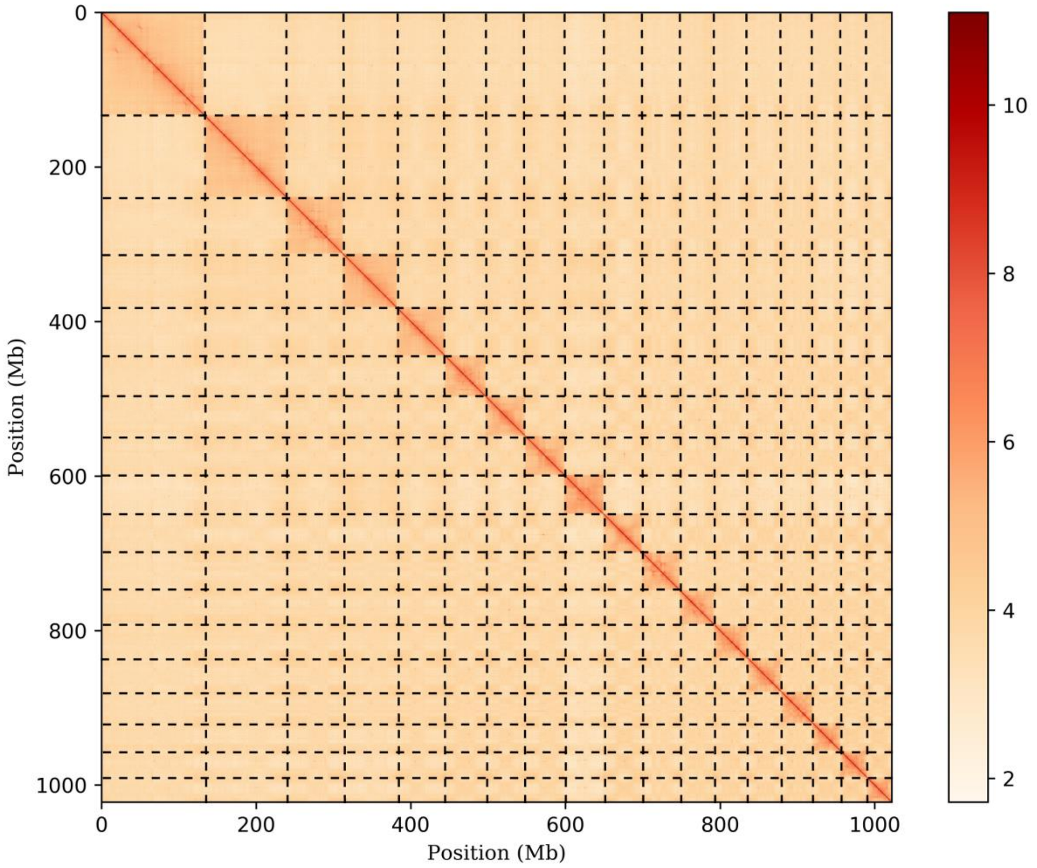

Figure S2 Note: Hi-C map showing genome-wide all-by-all interactions of the *GEE* genome and high resolution of 18 chromosomes scaffolds assembled independently.

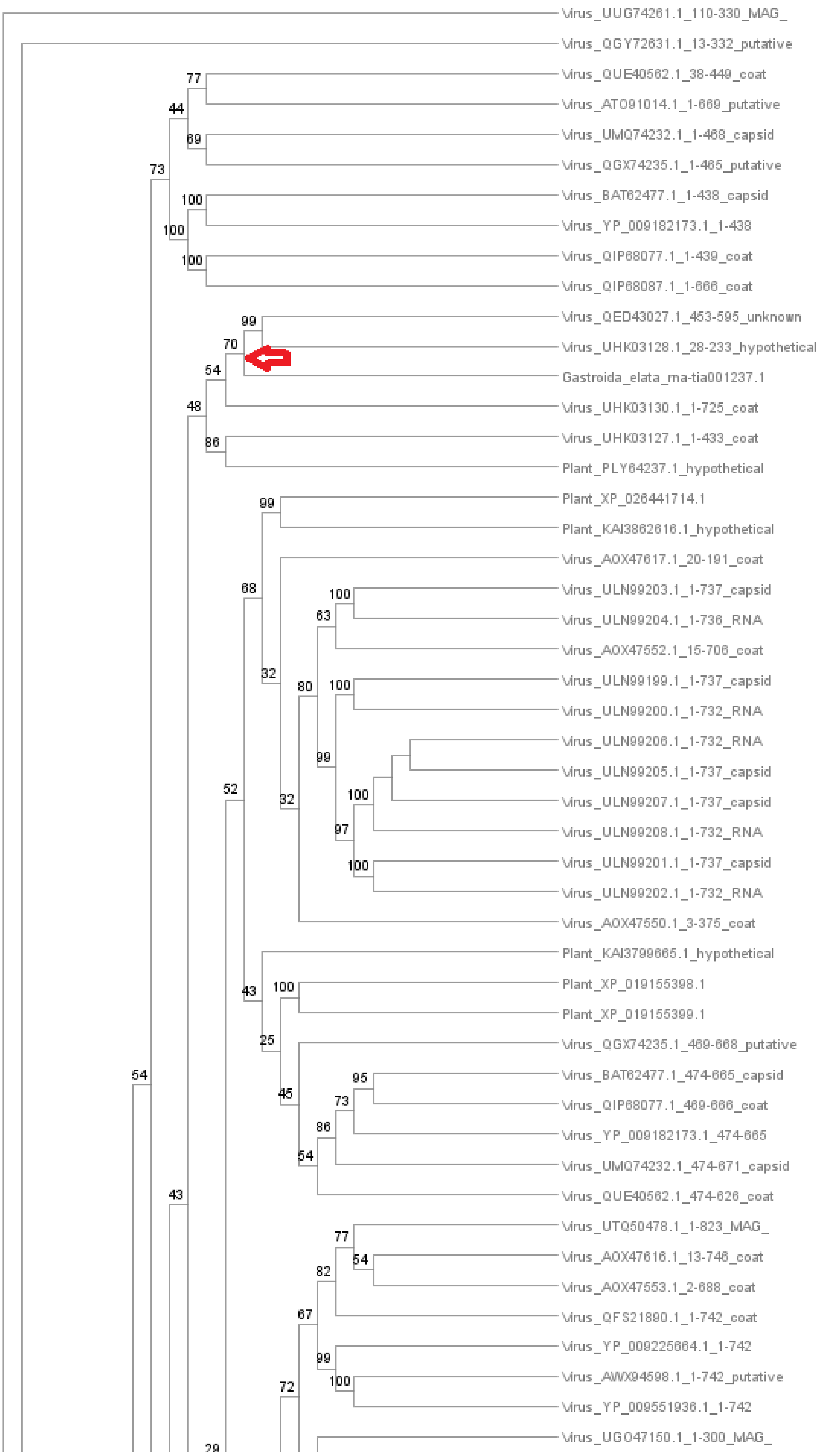
Virus_UUG74261.1_110-330_MAG_
Virus_QGY72631.1_13-332_putative
Virus_QUE40562.1_38-449_coat
Virus_ATO91014.1_1-669_putative
Virus_UMQ74232.1_1-468_capsid
Virus_QGX74235.1_1-465_putative
Virus_BAT62477.1_1-438_capsid
Virus_YP_009182173.1_1-438
Virus_QIP68077.1_1-439_coat
Virus_QIP68087.1_1-666_coat
Virus_QED43027.1_453-595_unknown
Virus_UHK03128.1_28-233_hypothetical
Gastroida_elata_rna-tia001237.1
Virus_UHK03130.1_1-725_coat
Virus_UHK03127.1_1-433_coat
Plant_PLY64237.1_hypothetical
Plant_XP_026441714.1
Plant_KAI3862616.1_hypothetical
Virus_AOX47617.1_20-191_coat
Virus_ULN99203.1_1-737_capsid
Virus_ULN99204.1_1-736_RNA
Virus_AOX47552.1_15-706_coat
Virus_ULN99199.1_1-737_capsid
Virus_ULN99200.1_1-732_RNA
Virus_ULN99206.1_1-732_RNA
Virus_ULN99205.1_1-737_capsid
Virus_ULN99207.1_1-737_capsid
Virus_ULN99208.1_1-732_RNA
Virus_ULN99201.1_1-737_capsid
Virus_ULN99202.1_1-732_RNA
Virus_AOX47550.1_3-375_coat
Plant_KAI3799665.1_hypothetical
Plant_XP_019155398.1
Plant_XP_019155399.1
Virus_QGX74235.1_469-668_putative
Virus_BAT62477.1_474-665_capsid
Virus_QIP68077.1_469-666_coat
Virus_YP_009182173.1_474-665
Virus_UMQ74232.1_474-671_capsid
Virus_QUE40562.1_474-626_coat
Virus_UTQ50478.1_1-823_MAG_
Virus_AOX47616.1_13-746_coat
Virus_AOX47553.1_2-688_coat
Virus_QFS21890.1_1-742_coat
Virus_YP_009225664.1_1-742
Virus_AWX94598.1_1-742_putative
Virus_YP_009551936.1_1-742
Virus_UGO47150.1_1-300_MAG_
77
44
69
73
100
100
100
99
70
54
48
86
99
68
100
63
32
80
100
99
52
32
100
97
100
43
100
25
45
95
73
86
54
54
43
77
54
82
67
99
100
72
29

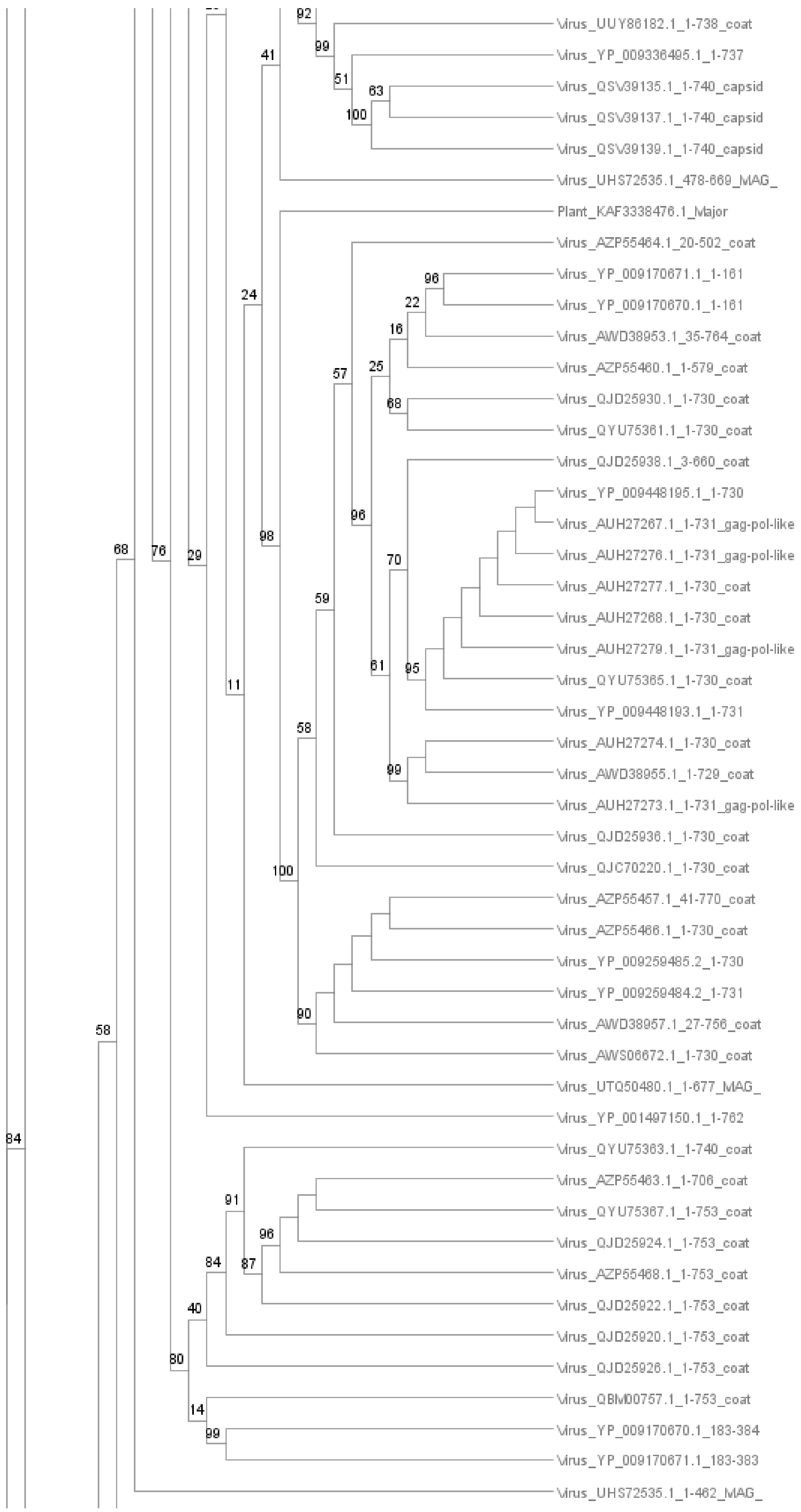

Virus_UUY86182.1_1-738_coat
Virus_YP_009336495.1_1-737
Virus_QSV39135.1_1-740_capsid
Virus_QSV39137.1_1-740_capsid
Virus_QSV39139.1_1-740_capsid
Virus_UHS72535.1_478-669_MAG_
Plant_KAF3338476.1_Major
Virus_AZP55464.1_20-502_coat
Virus_YP_009170671.1_1-161
Virus_YP_009170670.1_1-161
Virus_AWD38953.1_35-764_coat
Virus_AZP55460.1_1-579_coat
Virus_QJD25930.1_1-730_coat
Virus_QYU75361.1_1-730_coat
Virus_QJD25938.1_3-660_coat
Virus_YP_009448195.1_1-730
Virus_AUH27267.1_1-731_gag-pol-like
Virus_AUH27276.1_1-731_gag-pol-like
Virus_AUH27277.1_1-730_coat
Virus_AUH27268.1_1-730_coat
Virus_AUH27279.1_1-731_gag-pol-like
Virus_QYU75365.1_1-730_coat
Virus_YP_009448193.1_1-731
Virus_AUH27274.1_1-730_coat
Virus_AWD38955.1_1-729_coat
Virus_AUH27273.1_1-731_gag-pol-like
Virus_QJD25936.1_1-730_coat
Virus_QJC70220.1_1-730_coat
Virus_AZP55457.1_41-770_coat
Virus_AZP55466.1_1-730_coat
Virus_YP_009259485.2_1-730
Virus_YP_009259484.2_1-731
Virus_AWD38957.1_27-756_coat
Virus_AWS06672.1_1-730_coat
Virus_UTQ50480.1_1-677_MAG_
Virus_YP_001497150.1_1-762
Virus_QYU75363.1_1-740_coat
Virus_AZP55463.1_1-706_coat
Virus_QYU75367.1_1-753_coat
Virus_QJD25924.1_1-753_coat
Virus_AZP55468.1_1-753_coat
Virus_QJD25922.1_1-753_coat
Virus_QJD25920.1_1-753_coat
Virus_QJD25926.1_1-753_coat
Virus_QBM00757.1_1-753_coat
Virus_YP_009170670.1_183-384
Virus_YP_009170671.1_183-383
Virus_UHS72535.1_1-462_MAG_

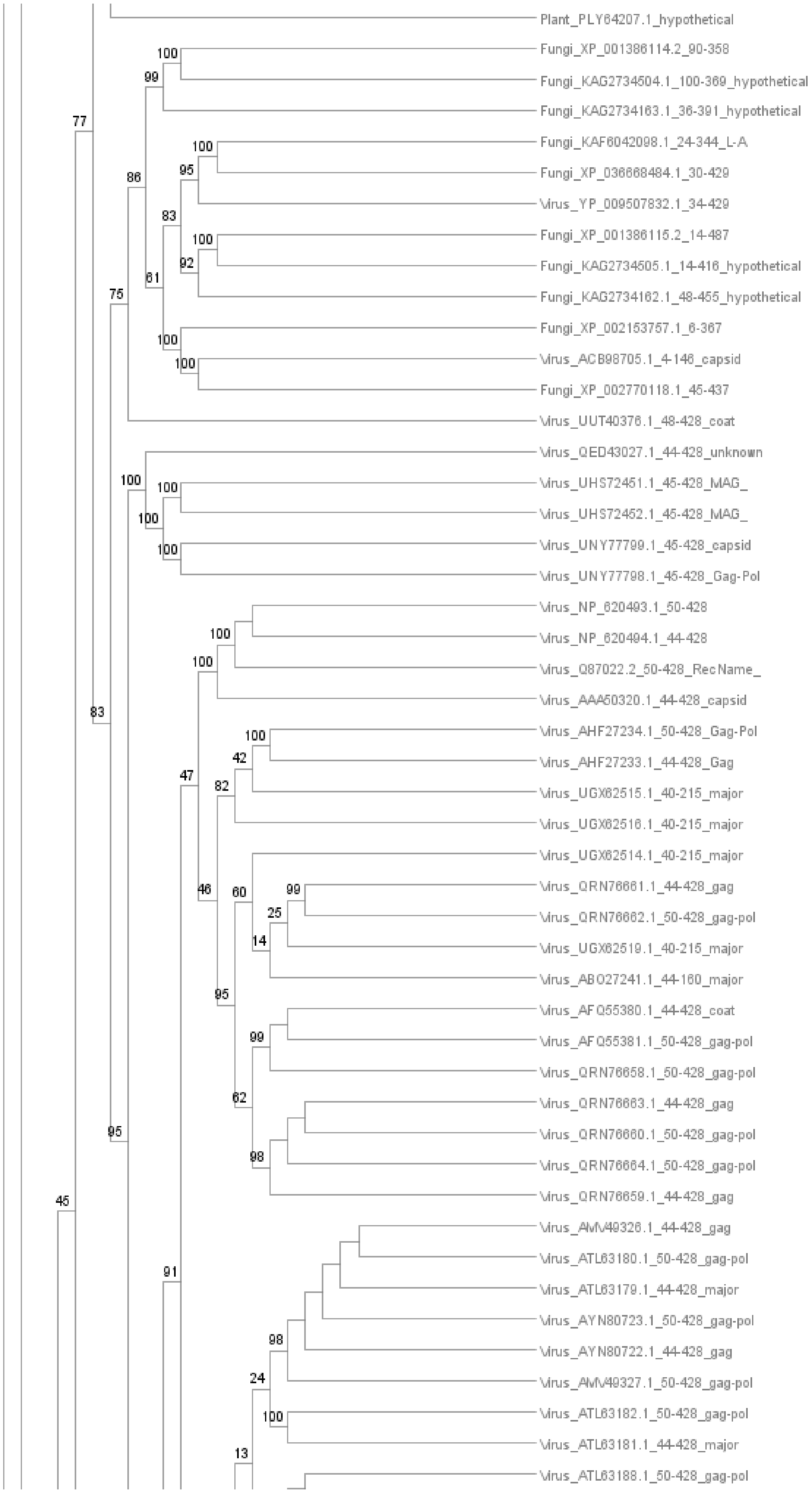

Plant_PLY64207.1_hypothetical
Fungi_XP_001386114.2_90-358
Fungi_KAG2734504.1_100-369_hypothetical
Fungi_KAG2734163.1_36-391_hypothetical
Fungi_KAF6042098.1_24-344_L-A
Fungi_XP_036668484.1_30-429
Virus_YP_009507832.1_34-429
Fungi_XP_001386115.2_14-487
Fungi_KAG2734505.1_14-416_hypothetical
Fungi_KAG2734162.1_48-455_hypothetical
Fungi_XP_002153757.1_6-367
Virus_ACB98705.1_4-146_capsid
Fungi_XP_002770118.1_45-437
Virus_UUT40376.1_48-428_coat
Virus_QED43027.1_44-428_unknown
Virus_UHS72451.1_45-428_MAG_
Virus_UHS72452.1_45-428_MAG_
Virus_UNY77799.1_45-428_capsid
Virus_UNY77798.1_45-428_Gag-Pol
Virus_NP_620493.1_50-428
Virus_NP_620494.1_44-428
Virus_Q87022.2_50-428_RecName_
Virus_AAA50320.1_44-428_capsid
Virus_AHF27234.1_50-428_Gag-Pol
Virus_AHF27233.1_44-428_Gag
Virus_UGX62515.1_40-215_major
Virus_UGX62516.1_40-215_major
Virus_UGX62514.1_40-215_major
Virus_QRN76661.1_44-428_gag
Virus_QRN76662.1_50-428_gag-pol
Virus_UGX62519.1_40-215_major
Virus_ABO27241.1_44-160_major
Virus_AFQ55380.1_44-428_coat
Virus_AFQ55381.1_50-428_gag-pol
Virus_QRN76658.1_50-428_gag-pol
Virus_QRN76663.1_44-428_gag
Virus_QRN76660.1_50-428_gag-pol
Virus_QRN76664.1_50-428_gag-pol
Virus_QRN76659.1_44-428_gag
Virus_AWV49326.1_44-428_gag
Virus_ATL63180.1_50-428_gag-pol
Virus_ATL63179.1_44-428_major
Virus_AYN80723.1_50-428_gag-pol
Virus_AYN80722.1_44-428_gag
Virus_AWV49327.1_50-428_gag-pol
Virus_ATL63182.1_50-428_gag-pol
Virus_ATL63181.1_44-428_major
Virus_ATL63188.1_50-428_gag-pol

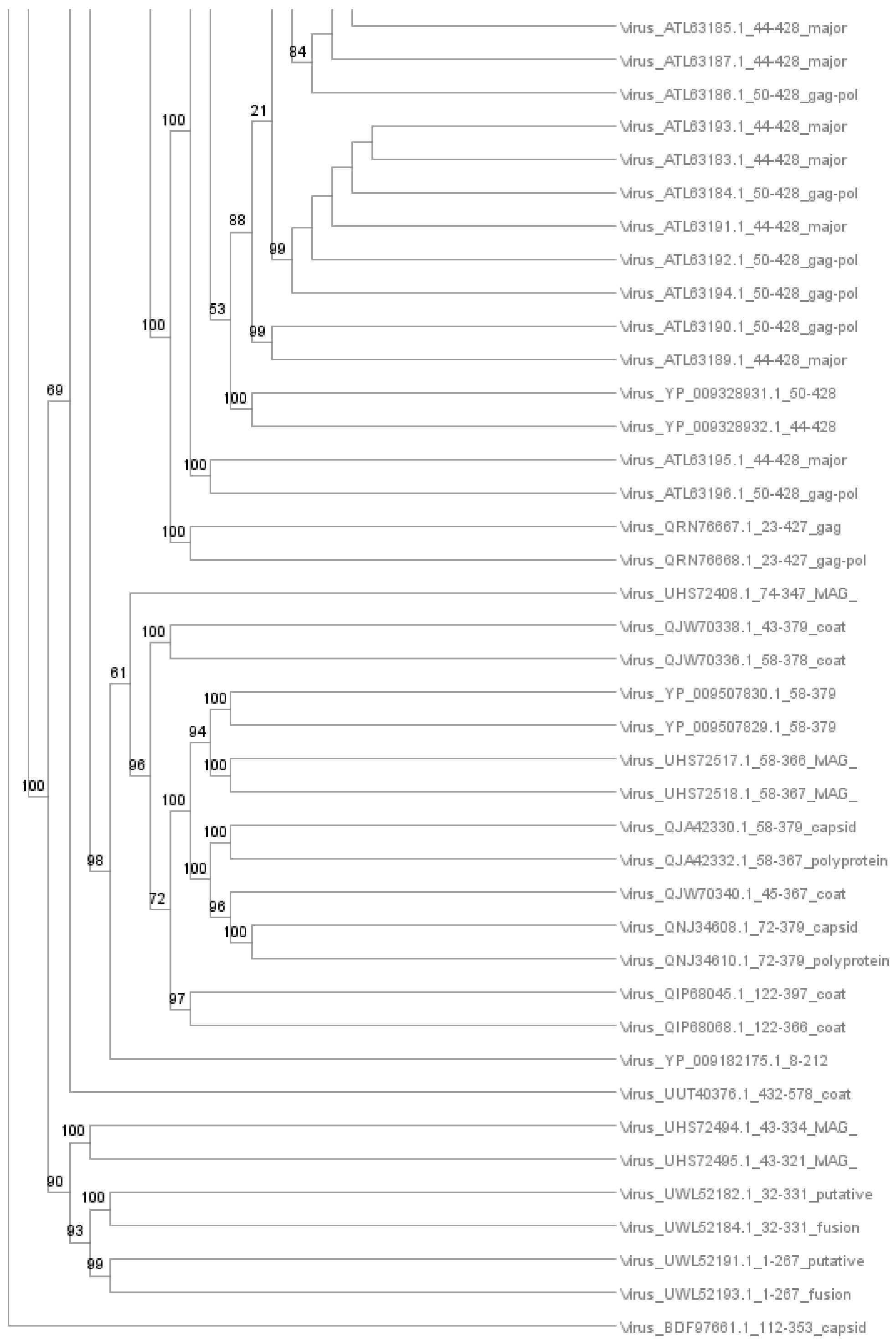

Figure S3 Phylogenetic tree constructed by whole homologs of rna-tia001237.1

Note: The red arrow indicates a HGT event related to *GE*

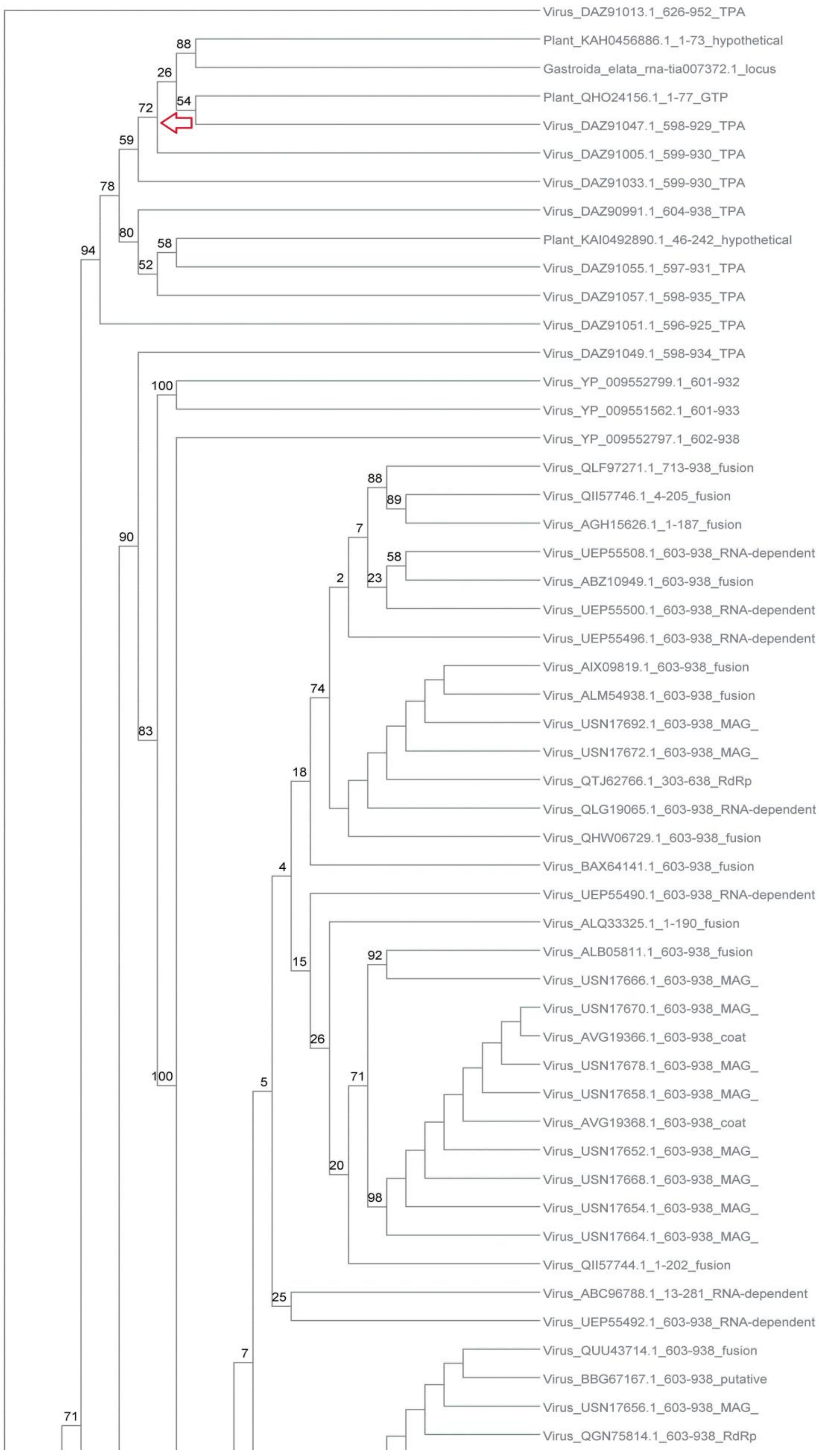
Virus_DAZ91013.1_626-952_TPA
Plant_KAH0456886.1_1-73_hypothetical
Gastroida_elata_rna-tia007372.1_locus
Plant_QHO24156.1_1-77_GTP
Virus_DAZ91047.1_598-929_TPA
Virus_DAZ91005.1_599-930_TPA
Virus_DAZ91033.1_599-930_TPA
Virus_DAZ90991.1_604-938_TPA
Plant_KAI0492890.1_46-242_hypothetical
Virus_DAZ91055.1_597-931_TPA
Virus_DAZ91057.1_598-935_TPA
Virus_DAZ91051.1_596-925_TPA
Virus_DAZ91049.1_598-934_TPA
Virus_YP_009552799.1_601-932
Virus_YP_009551562.1_601-933
Virus_YP_009552797.1_602-938
Virus_QLF97271.1_713-938_fusion
Virus_QII57746.1_4-205_fusion
Virus_AGH15626.1_1-187_fusion
Virus_UEP55508.1_603-938_RNA-dependent
Virus_ABZ10949.1_603-938_fusion
Virus_UEP55500.1_603-938_RNA-dependent
Virus_UEP55496.1_603-938_RNA-dependent
Virus_AIX09819.1_603-938_fusion
Virus_ALM54938.1_603-938_fusion
Virus_USN17692.1_603-938_MAG_
Virus_USN17672.1_603-938_MAG_
Virus_QTJ62766.1_303-638_RdRp
Virus_QLG19065.1_603-938_RNA-dependent
Virus_QHW06729.1_603-938_fusion
Virus_BAX64141.1_603-938_fusion
Virus_UEP55490.1_603-938_RNA-dependent
Virus_ALQ33325.1_1-190_fusion
Virus_ALB05811.1_603-938_fusion
Virus_USN17666.1_603-938_MAG_
Virus_USN17670.1_603-938_MAG_
Virus_AVG19366.1_603-938_coat
Virus_USN17678.1_603-938_MAG_
Virus_USN17658.1_603-938_MAG_
Virus_AVG19368.1_603-938_coat
Virus_USN17652.1_603-938_MAG_
Virus_USN17668.1_603-938_MAG_
Virus_USN17654.1_603-938_MAG_
Virus_USN17664.1_603-938_MAG_
Virus_QII57744.1_1-202_fusion
Virus_ABC96788.1_13-281_RNA-dependent
Virus_UEP55492.1_603-938_RNA-dependent
Virus_QUU43714.1_603-938_fusion
Virus_BBG67167.1_603-938_putative
Virus_USN17656.1_603-938_MAG_
Virus_QGN75814.1_603-938_RdRp
88
26
54
72
59
78
80
58
52
94
100
90
83
88
89
7
58
2
23
74
18
4
15
92
26
100
5
71
20
98
25
7
71

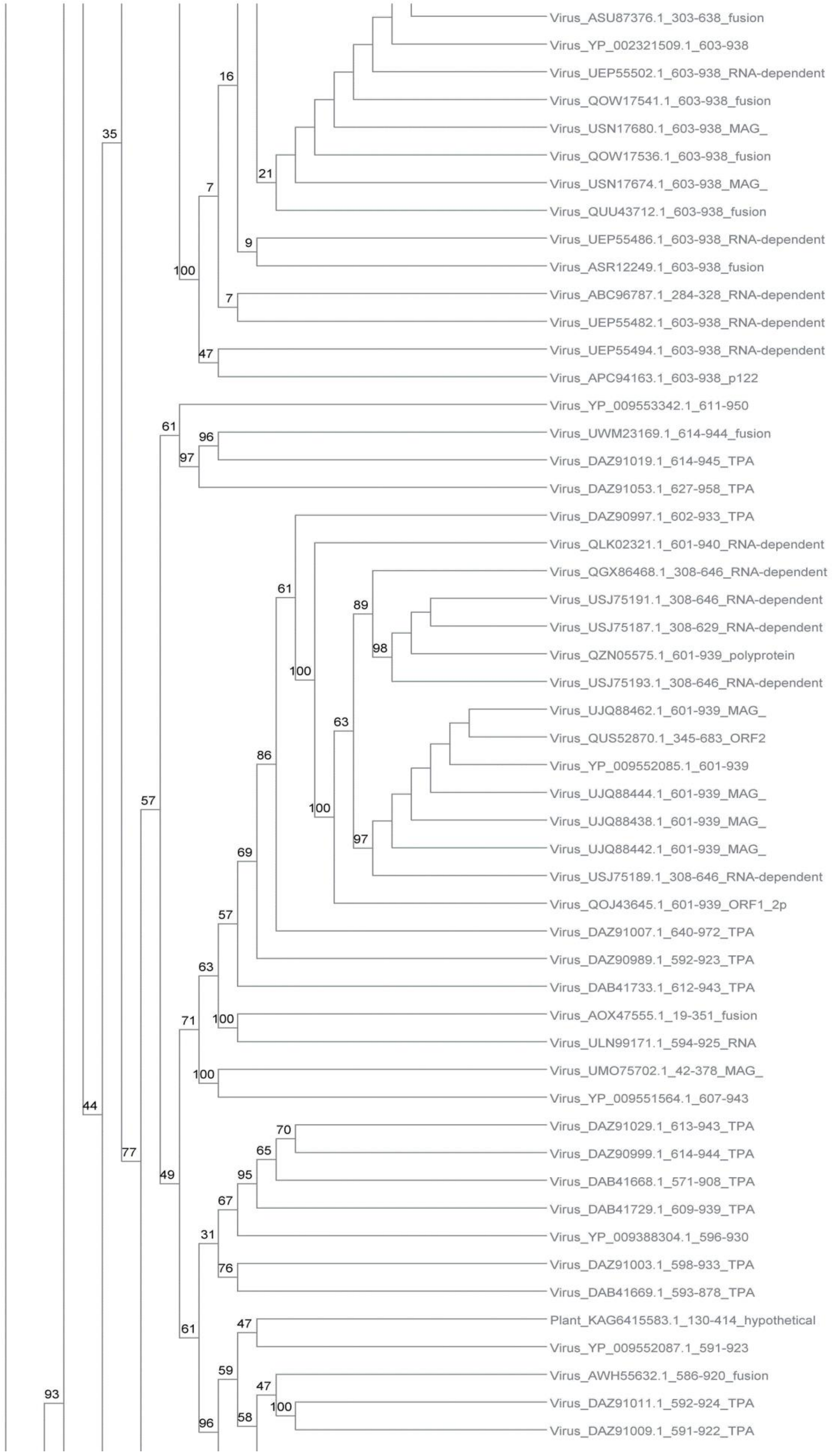
Virus_ASU87376.1_303-638_fusion
Virus_YP_002321509.1_603-938
Virus_UEP55502.1_603-938_RNA-dependent
Virus_QOW17541.1_603-938_fusion
Virus_USN17680.1_603-938_MAG_
Virus_QOW17536.1_603-938_fusion
Virus_USN17674.1_603-938_MAG_
Virus_QUU43712.1_603-938_fusion
Virus_UEP55486.1_603-938_RNA-dependent
Virus_ASR12249.1_603-938_fusion
Virus_ABC96787.1_284-328_RNA-dependent
Virus_UEP55482.1_603-938_RNA-dependent
Virus_UEP55494.1_603-938_RNA-dependent
Virus_APC94163.1_603-938_p122
Virus_YP_009553342.1_611-950
Virus_UWM23169.1_614-944_fusion
Virus_DAZ91019.1_614-945_TPA
Virus_DAZ91053.1_627-958_TPA
Virus_DAZ90997.1_602-933_TPA
Virus_QLK02321.1_601-940_RNA-dependent
Virus_QGX86468.1_308-646_RNA-dependent
Virus_USJ75191.1_308-646_RNA-dependent
Virus_USJ75187.1_308-629_RNA-dependent
Virus_QZN05575.1_601-939_polyprotein
Virus_USJ75193.1_308-646_RNA-dependent
Virus_UJQ88462.1_601-939_MAG_
Virus_QUS52870.1_345-683_ORF2
Virus_YP_009552085.1_601-939
Virus_UJQ88444.1_601-939_MAG_
Virus_UJQ88438.1_601-939_MAG_
Virus_UJQ88442.1_601-939_MAG_
Virus_USJ75189.1_308-646_RNA-dependent
Virus_QOJ43645.1_601-939_ORF1_2p
Virus_DAZ91007.1_640-972_TPA
Virus_DAZ90989.1_592-923_TPA
Virus_DAB41733.1_612-943_TPA
Virus_AOX47555.1_19-351_fusion
Virus_ULN99171.1_594-925_RNA
Virus_UMO75702.1_42-378_MAG_
Virus_YP_009551564.1_607-943
Virus_DAZ91029.1_613-943_TPA
Virus_DAZ90999.1_614-944_TPA
Virus_DAB41668.1_571-908_TPA
Virus_DAB41729.1_609-939_TPA
Virus_YP_009388304.1_596-930
Virus_DAZ91003.1_598-933_TPA
Virus_DAB41669.1_593-878_TPA
Plant_KAG6415583.1_130-414_hypothetical
Virus_YP_009552087.1_591-923
Virus_AWH55632.1_586-920_fusion
Virus_DAZ91011.1_592-924_TPA
Virus_DAZ91009.1_591-922_TPA

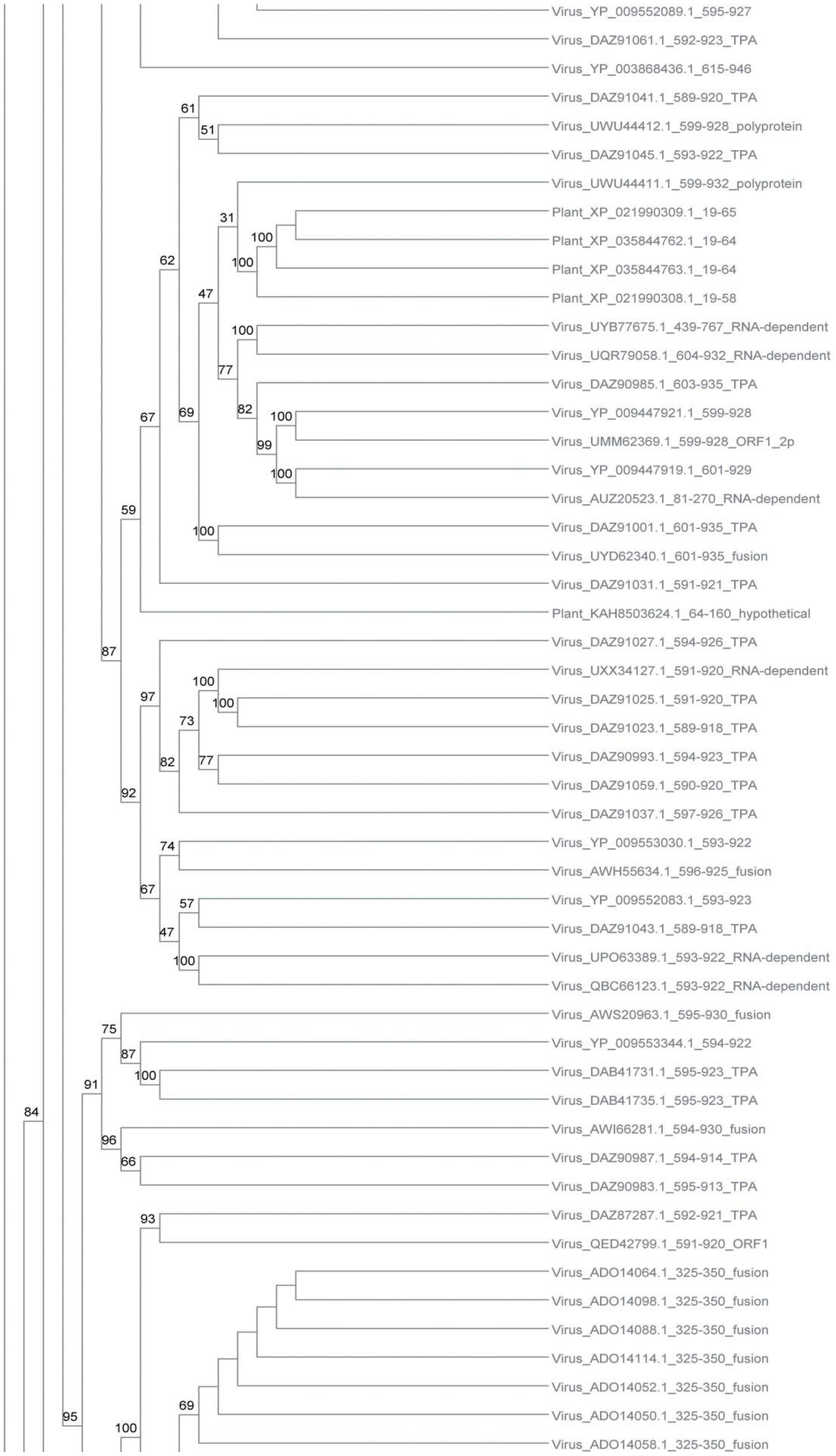
Virus_YP_009552089.1_595-927
Virus_DAZ91061.1_592-923_TPA
Virus_YP_003868436.1_615-946
Virus_DAZ91041.1_589-920_TPA
Virus_UWU44412.1_599-928_polyprotein
Virus_DAZ91045.1_593-922_TPA
Virus_UWU44411.1_599-932_polyprotein
Plant_XP_021990309.1_19-65
Plant_XP_035844762.1_19-64
Plant_XP_035844763.1_19-64
Plant_XP_021990308.1_19-58
Virus_UYB77675.1_439-767_RNA-dependent
Virus_UQR79058.1_604-932_RNA-dependent
Virus_DAZ90985.1_603-935_TPA
Virus_YP_009447921.1_599-928
Virus_UMM62369.1_599-928_ORF1_2p
Virus_YP_009447919.1_601-929
Virus_AUZ20523.1_81-270_RNA-dependent
Virus_DAZ91001.1_601-935_TPA
Virus_UYD62340.1_601-935_fusion
Virus_DAZ91031.1_591-921_TPA
Plant_KAH8503624.1_64-160_hypothetical
Virus_DAZ91027.1_594-926_TPA
Virus_UXX34127.1_591-920_RNA-dependent
Virus_DAZ91025.1_591-920_TPA
Virus_DAZ91023.1_589-918_TPA
Virus_DAZ90993.1_594-923_TPA
Virus_DAZ91059.1_590-920_TPA
Virus_DAZ91037.1_597-926_TPA
Virus_YP_009553030.1_593-922
Virus_AWH55634.1_596-925_fusion
Virus_YP_009552083.1_593-923
Virus_DAZ91043.1_589-918_TPA
Virus_UPO63389.1_593-922_RNA-dependent
Virus_QBC66123.1_593-922_RNA-dependent
Virus_AWS20963.1_595-930_fusion
Virus_YP_009553344.1_594-922
Virus_DAB41731.1_595-923_TPA
Virus_DAB41735.1_595-923_TPA
Virus_AWI66281.1_594-930_fusion
Virus_DAZ90987.1_594-914_TPA
Virus_DAZ90983.1_595-913_TPA
Virus_DAZ87287.1_592-921_TPA
Virus_QED42799.1_591-920_ORF1
Virus_ADO14064.1_325-350_fusion
Virus_ADO14098.1_325-350_fusion
Virus_ADO14088.1_325-350_fusion
Virus_ADO14114.1_325-350_fusion
Virus_ADO14052.1_325-350_fusion
Virus_ADO14050.1_325-350_fusion
Virus_ADO14058.1_325-350_fusion
61
51
31
100
100
62
47
100
77
82
100
67
69
99
100
59
100
87
100
97
100
73
82
77
92
74
67
57
47
100
75
87
100
91
84
96
66
93
69
95
100

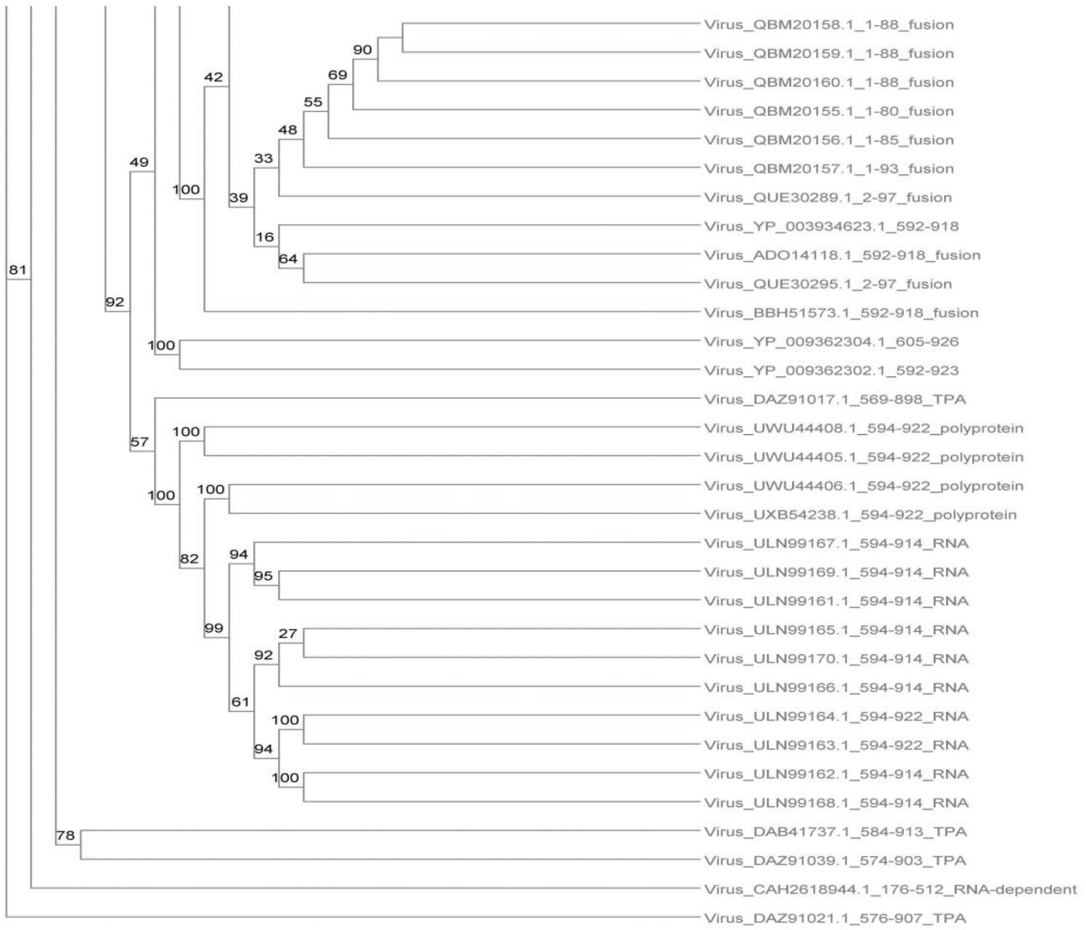

Figure S4 Phylogenetic tree constructed by whole homologs of rna-tia007273.1

Note: The red arrow indicates a HGT event related to *GE*

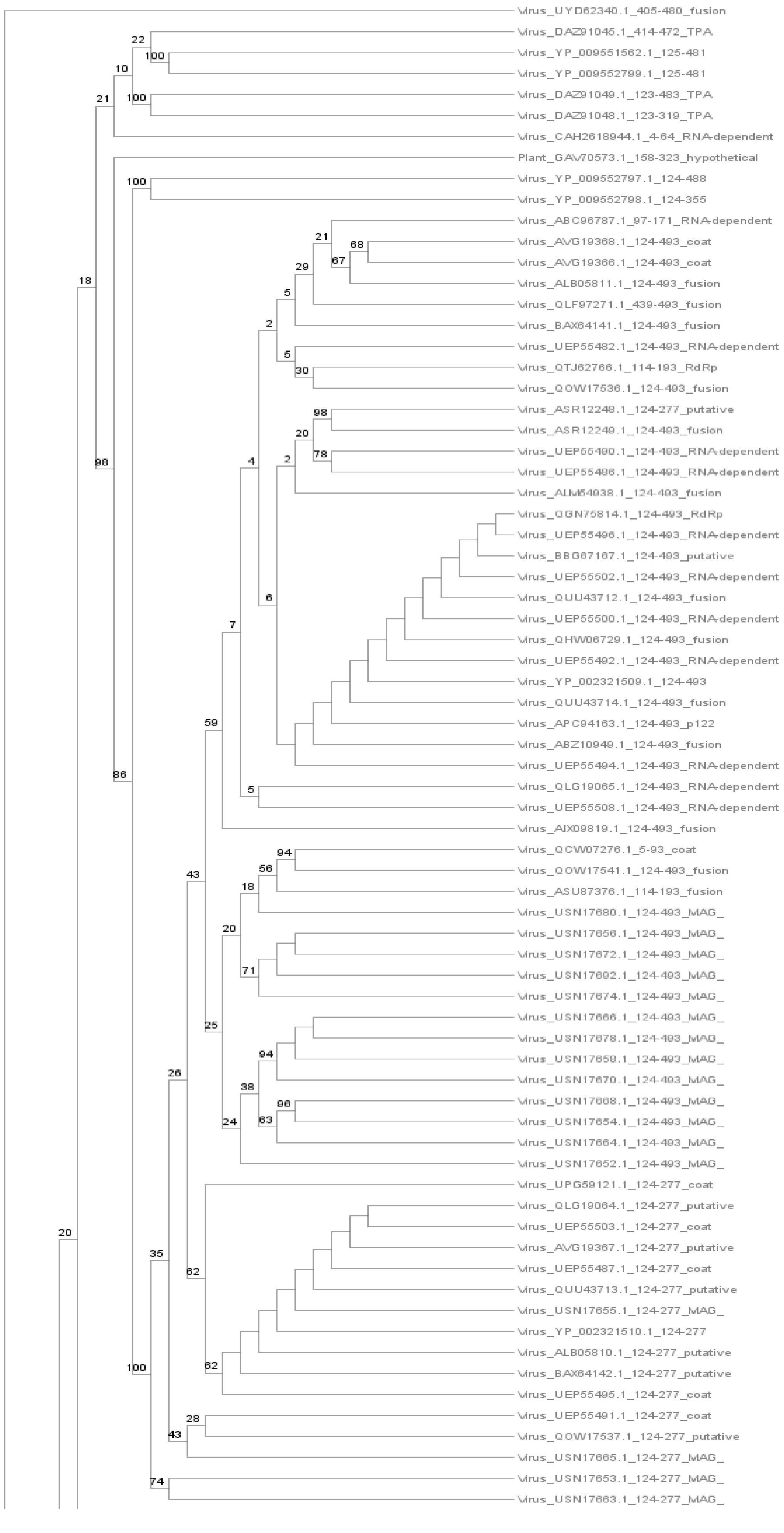

Virus_UYD62340.1_405-480_fusion
Virus_DAZ91045.1_414-472_TPA
Virus_YP_009551562.1_125-481
Virus_YP_009552799.1_125-481
Virus_DAZ91049.1_123-483_TPA
Virus_DAZ91048.1_123-319_TPA
Virus_CAH2618944.1_4-64_RNA-dependent
Plant_GAV70573.1_158-323_hypothetical
Virus_YP_009552797.1_124-488
Virus_YP_009552798.1_124-355
Virus_ABC96787.1_97-171_RNA-dependent
Virus_AVG19368.1_124-493_coat
Virus_AVG19366.1_124-493_coat
Virus_ALB05811.1_124-493_fusion
Virus_QLF97271.1_439-493_fusion
Virus_BAX64141.1_124-493_fusion
Virus_UEP55482.1_124-493_RNA-dependent
Virus_QTJ62766.1_114-193_RdRp
Virus_QOW17536.1_124-493_fusion
Virus_ASR12248.1_124-277_putative
Virus_ASR12249.1_124-493_fusion
Virus_UEP55490.1_124-493_RNA-dependent
Virus_UEP55486.1_124-493_RNA-dependent
Virus_ALM54938.1_124-493_fusion
Virus_QGN75814.1_124-493_RdRp
Virus_UEP55496.1_124-493_RNA-dependent
Virus_BBG67167.1_124-493_putative
Virus_UEP55502.1_124-493_RNA-dependent
Virus_QUU43712.1_124-493_fusion
Virus_UEP55500.1_124-493_RNA-dependent
Virus_QHW06729.1_124-493_fusion
Virus_UEP55492.1_124-493_RNA-dependent
Virus_YP_002321509.1_124-493
Virus_QUU43714.1_124-493_fusion
Virus_APC94163.1_124-493_p122
Virus_ABZ10949.1_124-493_fusion
Virus_UEP55494.1_124-493_RNA-dependent
Virus_QLG19065.1_124-493_RNA-dependent
Virus_UEP55508.1_124-493_RNA-dependent
Virus_AIX09819.1_124-493_fusion
Virus_QCW07276.1_5-93_coat
Virus_QOW17541.1_124-493_fusion
Virus_ASU87376.1_114-193_fusion
Virus_USN17680.1_124-493_MAG_
Virus_USN17656.1_124-493_MAG_
Virus_USN17672.1_124-493_MAG_
Virus_USN17692.1_124-493_MAG_
Virus_USN17674.1_124-493_MAG_
Virus_USN17666.1_124-493_MAG_
Virus_USN17678.1_124-493_MAG_
Virus_USN17658.1_124-493_MAG_
Virus_USN17670.1_124-493_MAG_
Virus_USN17668.1_124-493_MAG_
Virus_USN17654.1_124-493_MAG_
Virus_USN17664.1_124-493_MAG_
Virus_USN17652.1_124-493_MAG_
Virus_UPG59121.1_124-277_coat
Virus_QLG19064.1_124-277_putative
Virus_UEP55503.1_124-277_coat
Virus_AVG19367.1_124-277_putative
Virus_UEP55487.1_124-277_coat
Virus_QUU43713.1_124-277_putative
Virus_USN17655.1_124-277_MAG_
Virus_YP_002321510.1_124-277
Virus_ALB05810.1_124-277_putative
Virus_BAX64142.1_124-277_putative
Virus_UEP55495.1_124-277_coat
Virus_UEP55491.1_124-277_coat
Virus_QOW17537.1_124-277_putative
Virus_USN17665.1_124-277_MAG_
Virus_USN17653.1_124-277_MAG_
Virus_USN17663.1_124-277_MAG_

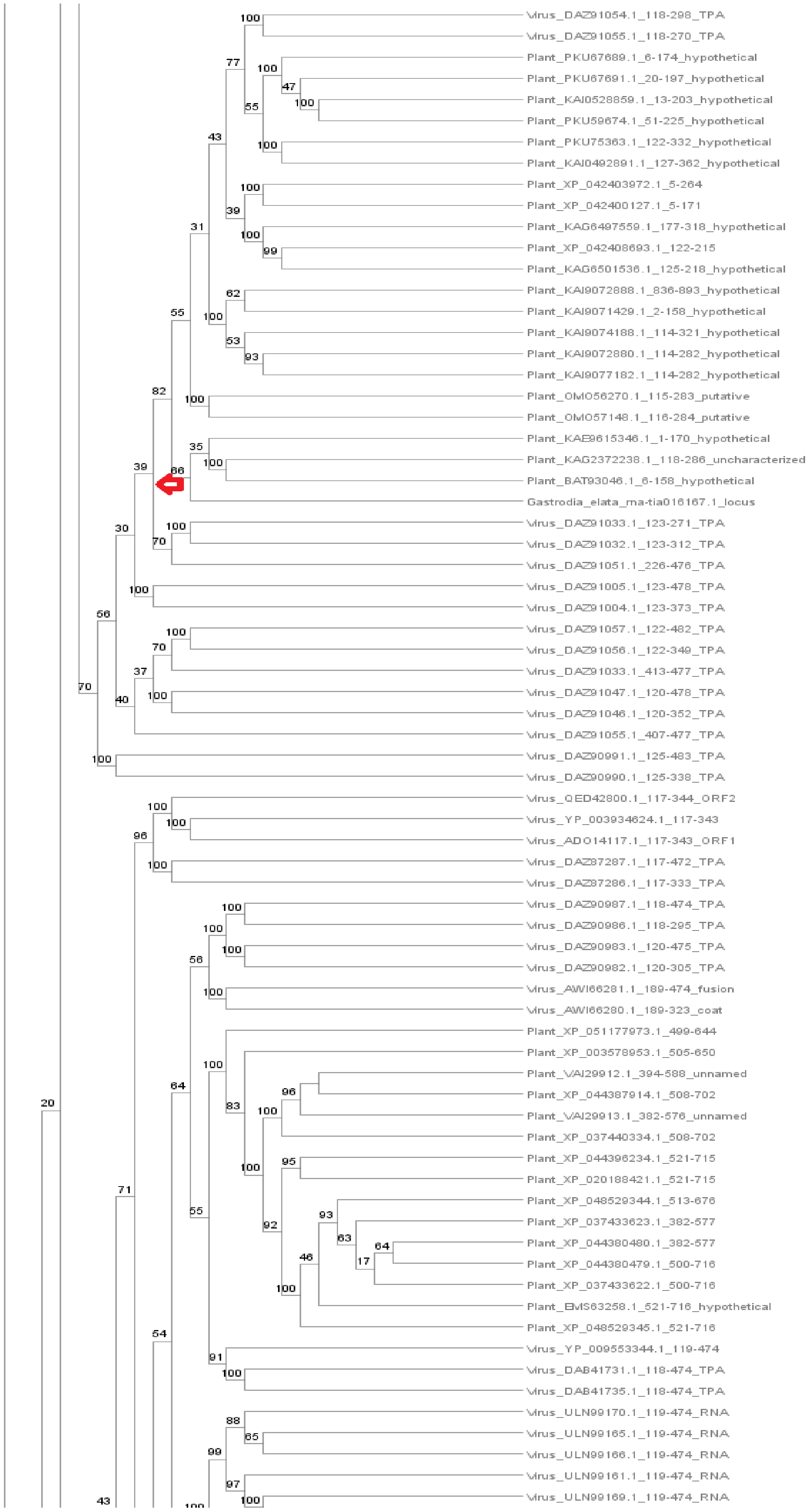

Virus_DAZ91054.1_118-298_TPA
Virus_DAZ91055.1_118-270_TPA
Plant_PKU67689.1_6-174_hypothetical
Plant_PKU67691.1_20-197_hypothetical
Plant_KAI0528859.1_13-203_hypothetical
Plant_PKU59674.1_51-225_hypothetical
Plant_PKU75363.1_122-332_hypothetical
Plant_KAI0492891.1_127-362_hypothetical
Plant_XP_042403972.1_5-264
Plant_XP_042400127.1_5-171
Plant_KAG6497559.1_177-318_hypothetical
Plant_XP_042408693.1_122-215
Plant_KAG6501536.1_125-218_hypothetical
Plant_KAI9072888.1_836-893_hypothetical
Plant_KAI9071429.1_2-158_hypothetical
Plant_KAI9074188.1_114-321_hypothetical
Plant_KAI9072880.1_114-282_hypothetical
Plant_KAI9077182.1_114-282_hypothetical
Plant_OMO56270.1_115-283_putative
Plant_OMO57148.1_116-284_putative
Plant_KAE9615346.1_1-170_hypothetical
Plant_KAG2372238.1_118-286_uncharacterized
Plant_BAT93046.1_6-158_hypothetical
Gastrodia_elata_rna-tia016167.1_locus
Virus_DAZ91033.1_123-271_TPA
Virus_DAZ91032.1_123-312_TPA
Virus_DAZ91051.1_226-476_TPA
Virus_DAZ91005.1_123-478_TPA
Virus_DAZ91004.1_123-373_TPA
Virus_DAZ91057.1_122-482_TPA
Virus_DAZ91056.1_122-349_TPA
Virus_DAZ91033.1_413-477_TPA
Virus_DAZ91047.1_120-478_TPA
Virus_DAZ91046.1_120-352_TPA
Virus_DAZ91055.1_407-477_TPA
Virus_DAZ90991.1_125-483_TPA
Virus_DAZ90990.1_125-338_TPA
Virus_QED42800.1_117-344_ORF2
Virus_YP_003934624.1_117-343
Virus_ADO14117.1_117-343_ORF1
Virus_DAZ87287.1_117-472_TPA
Virus_DAZ87286.1_117-333_TPA
Virus_DAZ90987.1_118-474_TPA
Virus_DAZ90986.1_118-295_TPA
Virus_DAZ90983.1_120-475_TPA
Virus_DAZ90982.1_120-305_TPA
Virus_AWI66281.1_189-474_fusion
Virus_AWI66280.1_189-323_coat
Plant_XP_051177973.1_499-644
Plant_XP_003578953.1_505-650
Plant_VAI29912.1_394-588_unnamed
Plant_XP_044387914.1_508-702
Plant_VAI29913.1_382-576_unnamed
Plant_XP_037440334.1_508-702
Plant_XP_044396234.1_521-715
Plant_XP_020188421.1_521-715
Plant_XP_048529344.1_513-676
Plant_XP_037433623.1_382-577
Plant_XP_044380480.1_382-577
Plant_XP_044380479.1_500-716
Plant_XP_037433622.1_500-716
Plant_EMS63258.1_521-716_hypothetical
Plant_XP_048529345.1_521-716
Virus_YP_009553344.1_119-474
Virus_DAB41731.1_118-474_TPA
Virus_DAB41735.1_118-474_TPA
Virus_ULN99170.1_119-474_RNA
Virus_ULN99165.1_119-474_RNA
Virus_ULN99166.1_119-474_RNA
Virus_ULN99161.1_119-474_RNA
Virus_ULN99169.1_119-474_RNA

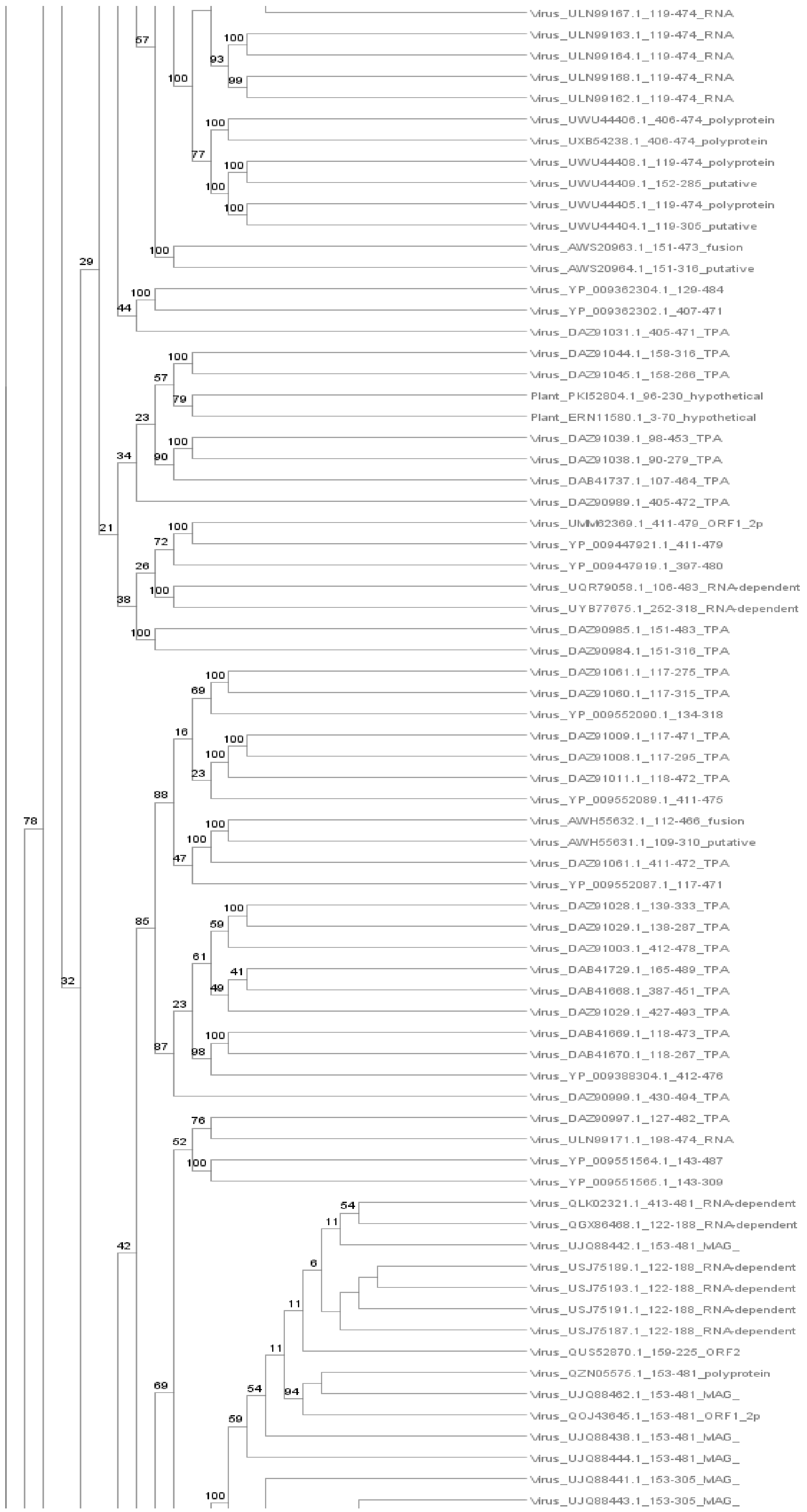
Virus_ULN99167.1_119-474_RNA
Virus_ULN99163.1_119-474_RNA
Virus_ULN99164.1_119-474_RNA
Virus_ULN99168.1_119-474_RNA
Virus_ULN99162.1_119-474_RNA
Virus_UWU44406.1_406-474_polyprotein
Virus_UXB54238.1_406-474_polyprotein
Virus_UWU44408.1_119-474_polyprotein
Virus_UWU44409.1_152-285_putative
Virus_UWU44405.1_119-474_polyprotein
Virus_UWU44404.1_119-305_putative
Virus_AWS20963.1_151-473_fusion
Virus_AWS20964.1_151-316_putative
Virus_YP_009362304.1_129-484
Virus_YP_009362302.1_407-471
Virus_DAZ91031.1_405-471_TPA
Virus_DAZ91044.1_158-316_TPA
Virus_DAZ91045.1_158-266_TPA
Plant_PKI52804.1_96-230_hypothetical
Plant_ERN11580.1_3-70_hypothetical
Virus_DAZ91039.1_98-453_TPA
Virus_DAZ91038.1_90-279_TPA
Virus_DAB41737.1_107-464_TPA
Virus_DAZ90989.1_405-472_TPA
Virus_UMM62369.1_411-479_ORF1_2p
Virus_YP_009447921.1_411-479
Virus_YP_009447919.1_397-480
Virus_UQR79058.1_106-483_RNA-dependent
Virus_UYB77675.1_252-318_RNA-dependent
Virus_DAZ90985.1_151-483_TPA
Virus_DAZ90984.1_151-316_TPA
Virus_DAZ91061.1_117-275_TPA
Virus_DAZ91060.1_117-315_TPA
Virus_YP_009552090.1_134-318
Virus_DAZ91009.1_117-471_TPA
Virus_DAZ91008.1_117-295_TPA
Virus_DAZ91011.1_118-472_TPA
Virus_YP_009552089.1_411-475
Virus_AWH55632.1_112-466_fusion
Virus_AWH55631.1_109-310_putative
Virus_DAZ91061.1_411-472_TPA
Virus_YP_009552087.1_117-471
Virus_DAZ91028.1_139-333_TPA
Virus_DAZ91029.1_138-287_TPA
Virus_DAZ91003.1_412-478_TPA
Virus_DAB41729.1_165-489_TPA
Virus_DAB41668.1_387-451_TPA
Virus_DAZ91029.1_427-493_TPA
Virus_DAB41669.1_118-473_TPA
Virus_DAB41670.1_118-267_TPA
Virus_YP_009388304.1_412-476
Virus_DAZ90999.1_430-494_TPA
Virus_DAZ90997.1_127-482_TPA
Virus_ULN99171.1_198-474_RNA
Virus_YP_009551564.1_143-487
Virus_YP_009551565.1_143-309
Virus_QLK02321.1_413-481_RNA-dependent
Virus_QGX86468.1_122-188_RNA-dependent
Virus_UJQ88442.1_153-481_MAG_
Virus_USJ75189.1_122-188_RNA-dependent
Virus_USJ75193.1_122-188_RNA-dependent
Virus_USJ75191.1_122-188_RNA-dependent
Virus_USJ75187.1_122-188_RNA-dependent
Virus_QUS52870.1_159-225_ORF2
Virus_QZN05575.1_153-481_polyprotein
Virus_UJQ88462.1_153-481_MAG_
Virus_QOJ43645.1_153-481_ORF1_2p
Virus_UJQ88438.1_153-481_MAG_
Virus_UJQ88444.1_153-481_MAG_
Virus_UJQ88441.1_153-305_MAG_
Virus_UJQ88443.1_153-305_MAG_

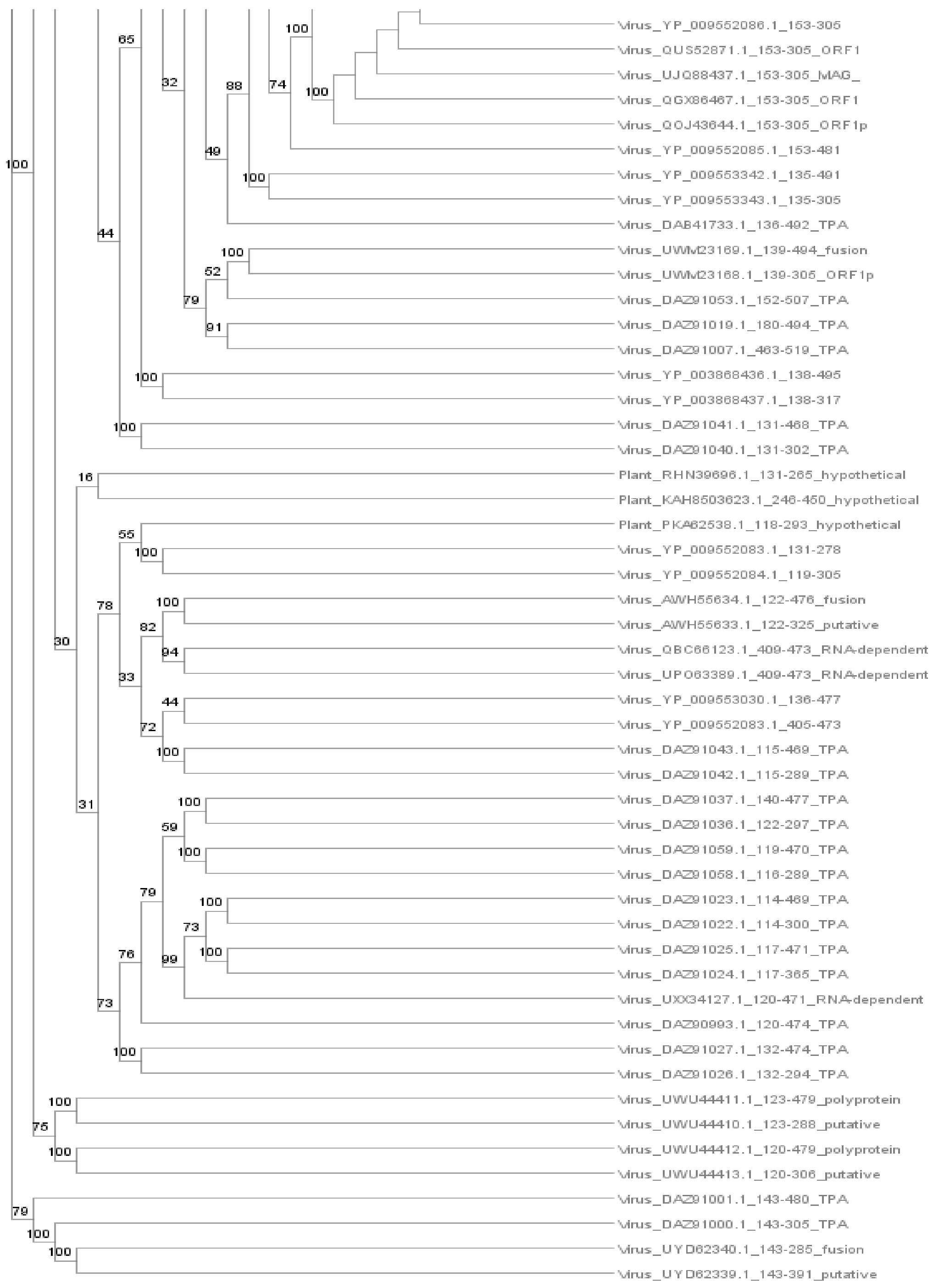

Figure S5 Phylogenetic tree constructed by whole homologous sequence of rna-tia016167.1

Note: The red arrow indicates a HGT event related to *GE*

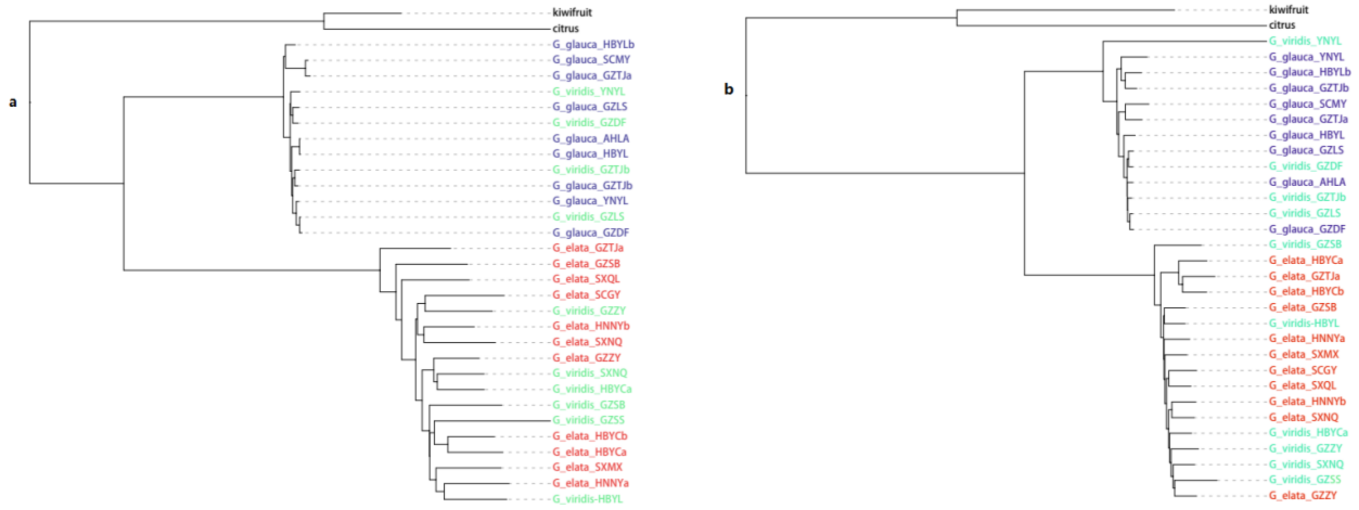

Figure S6 Phylogenetic tree of all samples constructed by cytoplasmic genome: a, chloroplast tree; b, mitochondria tree

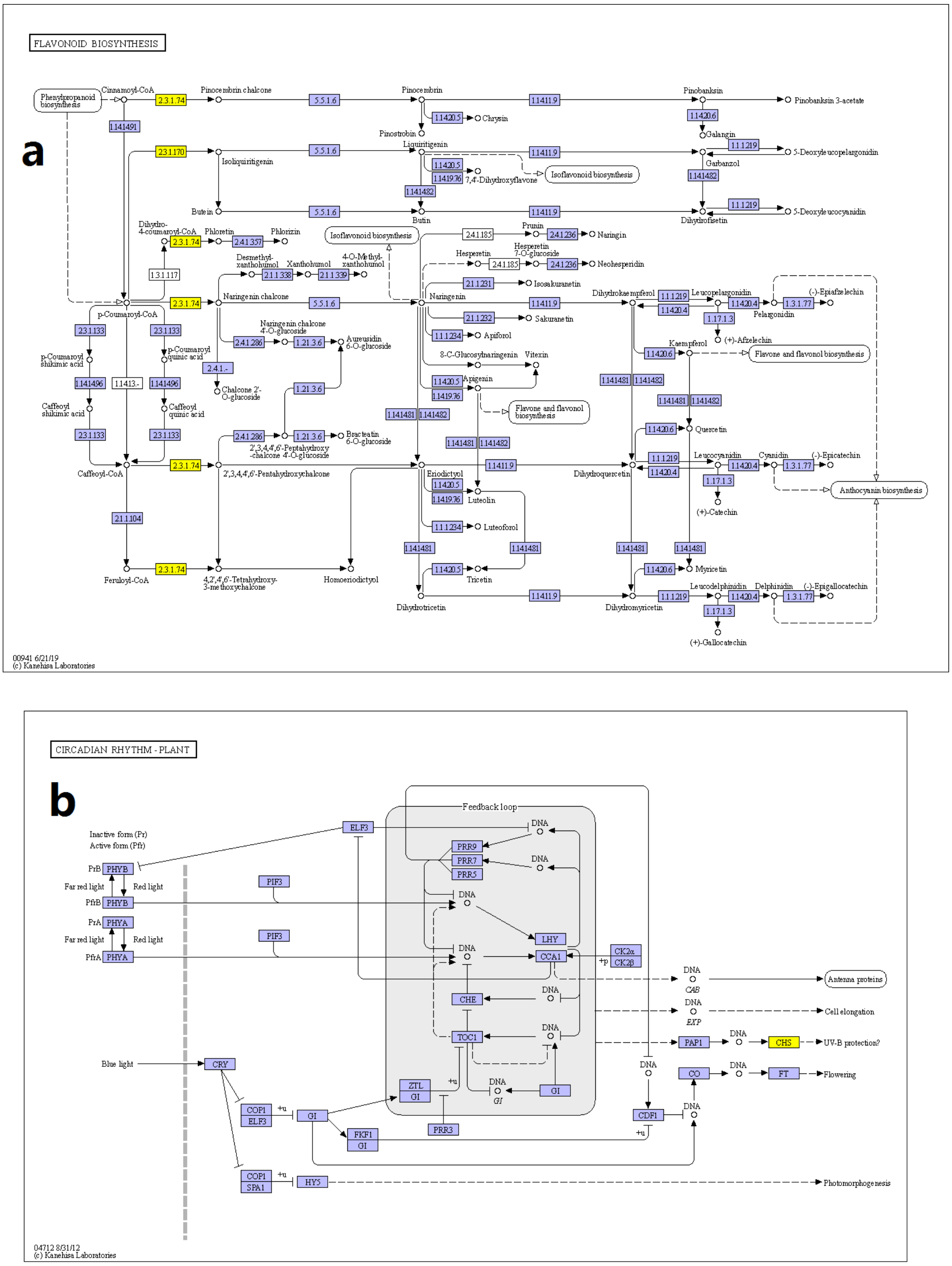

Figure S7 KEGG pathway maps: a, the flavonoid biosynthesis pathway; b, circadian rhythm-plant pathway

Note: Enzyme compound in yellow box indicated the chalcone synthase encoded by genes tia000401, tia000402, and tia000403

Table S1 Sampling information for sequencing and re-sequencing of 30 *GE* accessions

Sample Code	Sampling Name	Sampling Area	Sample Source	Varieties Form
G01	*G_viridis*_YNYL	Yiliang county, Yunnan province	In cultivated population, donored by Rui Wang	*G. elata* Bl. f. viridis
G02	*G_glauca*_YNYL	Yiliang county, Yunnan province	In the mountain forest, N26°28′56″, E103°51′34″	*G. elata* Bl. f. glauca
G03	*G_elata*_HBYCa	Yichang city, Hubei province	Bought from local market	*G. elata* Bl. f. elata
G04	*G_viridis*_HBYCa	Yichang city, Hubei province	In cultivated population, donored by Xiaojun Chen	*G. elata* Bl. f. viridis
G05	*G_elata*_GZZY	Zhenyuan county, Guizhou province	In the mountain forest, N27°03′45″,108°26′12″	*G. elata* Bl. f. elata
G06	*G_viridis*_GZZY	Zhenyuan county, Guizhou province	In cultivated population, donored by Heng Wang	*G. elata* Bl. f. viridis
G07	*G_elata*_SXNQ	Ningqiang county, Shanaxi province	In the mountain forest, N30°16′28″, E111°11′59″	*G. elata* Bl. f. elata
G08	*G_viridis*_SXNQ	Ningqiang county, Shanaxi province	In cultivated population, donored by Dali Huang	*G. elata* Bl. f. viridis
G09	*G_viridis*-HBYL	Yiling county, Hubei province	In cultivated population, donored by Juan Zhao	*G. elata* Bl. f. viridis
G10	*G_elata*_SXQL	Qinling county, Shanaxi province	In the mountain forest, N32′46.13″, E108°37′07″	*G. elata* Bl. f. elata
G11	*G_glauca*_HBYL	Yiling county, Hubei province	In the mountain forest, N30°36′05″, E111°20′30″	*G. elata* Bl. f. glauca
G12	*G_elata*_HBYCb	Yichang city, Hubei province	In the mountain forest, N30°48′37″, E111°22′09″	*G. elata* Bl. f. elata
G13	*G_elata*_HNNYa	Nanyang county, Henan province	In the mountain forest, N34°38′15″, E112°19′11″	*G. elata* Bl. f. elata
G14	*G_elata*_SXMX	Mianxian county, Shanaxi province	In the mountain forest, N33°05′42″, E106°27′38″	*G. elata* Bl. f. elata
G15	*G_elata*_HNNYb	Nanyang county, Henan province	In the mountain forest, N33°38′53″, E112°31′17″	*G. elata* Bl. f. elata
G16	*G_glauca*_AHLA	Liuan county, Anhui province	In the mountain forest, N31°43′08″, E116°30′18″	*G. elata* Bl. f. glauca
G17	*G_elata*_SCGY	Guangyuan county, Sichuan province	In the mountain forest, N32°54′33″, E109°59′48″	*G. elata* Bl. f. elata
G18	*G_elata*_GZTJa	Taijiang county, Guizhou province	In the mountain forest, N26°37′45″, E108°18′53″	*G. elata* Bl. f. elata
G19	*G_glauca*_GZTJa	Taijiang county, Guizhou province	In the mountain forest, N26°37′42″, E108°18′39″	*G. elata* Bl. f. glauca

G20	*G_viridis*_GZDF	Dafang county, Guizhou province	In cultivated population, donored by Guangwen Zhang	*G. elata* Bl. f. viridis
G21	*G_glauca*_GZDF	Dafang county, Guizhou province	In the mountain forest, N26°09′31″, E105°36′12″	*G. elata* Bl. f. glauca
G22	*G_elata*_GZSB	Shibing county, Guizhou province	In the mountain forest, N27°01′37″, E108°12′52″	*G. elata* Bl. f. elata
G23	*G_viridis*_GZSB	Shibing county, Guizhou province	In cultivated population, donored by Youming Li	*G. elata* Bl. f. viridis
G24	*G_viridis*_GZSS	Sansui county, Guizhou province	In cultivated population, donored by Jingwu Liu	*G. elata* Bl. f. viridis
G25	*G_glauca*_GZTJb	Taijiang county, Guizhou province	In the mountain forest, N26°37′19″, E108°18′46″	*G. elata* Bl. f. glauca
G26	*G_viridis*_GZTJb	Taijiang county, Guizhou province	In cultivated population, donored by Xiaohong Liu	*G. elata* Bl. f. viridis
G27	*G_glauca*_SCMY	Mianyang county, Sichuan province	In the mountain forest, N31°46′08″, E104°55′09″	*G. elata* Bl. f. glauca
G28	*G_glauca*_HBYLb	Yiling county, Hubei province	In the mountain forest, N30°42′24″, E111°21′29″	*G. elata* Bl. f. glauca
G29	*G_glauca*_GZLS	Leishan county, Hubei province	In the mountain forest, N26°39′06″, E108°08′18″	*G. elata* Bl. f. glauca
G30	*G_viridis*_GZLS	Leishan county, Hubei province	In cultivated population, donored by Guisheng Tao	*G. elata* Bl. f. viridis

Table S2 Statistics of short-reads data from G03 generated by MGISEQ-2000 platform

Items	Counts		
	G03_1-1 Library	G03_1-2 Library	Combination
Read Number	180,003,082	180,003,082	360,006,164
Base Count (bp)	26,820,646,891	26,715,977,358	53,536,624,249
Read Length (bp)	149	148	NA
Q20 (%)	98.4	97.6	NA
Q30 (%)	94.5	92.2	NA

NA indicates not applicable

Table S3 K-mer statistics of short-reads data from G03 generated by Illumina Hiseq 4000

Items	Counts
K-mer score	17
K-mer number	47,179,493,375
K-mer Depth	42
Genome Size (Mb)	1,086.79
Heterozygous Ratio (%)	0.33
Repeat (%)	63.89

Table S4 Statistics of long-reads data of G03 generated by PioBac

Items	Counts
Total bases (bp)	130,491,707,713
Subread number	7,672,586
Average subread length (bp)	17,007
Subread length N50 (bp)	26,754

Table S5 Assembly statistics of contigs and scaffolds of G03 draft genome

Name	Scaffold Length (bp)	Scaffold Number	Contig Length (bp)	Contig Number
max_len	133,096,707	-	64,326,117	-
N10	133,096,707	1	49,033,157	2
N20	105,625,784	2	34,247,810	5
N30	73,576,681	3	24,160,711	9
N40	62,506,212	5	20,437,881	13
N50	51,201,707	7	16,871,000	19
N60	50,298,580	9	13,433,884	26
N70	48,640,294	11	11,209,081	35
N80	43,675,728	13	7,960,202	45
N90	36,291,072	16	4,958,000	62
Total_length	1,036,918,901	475	1,036,828,901	655

Table S6 Assembly evaluation of G03 draft genome by nucleotides statistics

Nucleotides	Length (bp)	% of genome
A	340,583,051	32.85
C	177,753,458	17.14
G	177,783,088	17.15
T	340,709,304	32.86
N	90,000	0.01
G+C	355,536,546	34.29

Table S7 Assembly evaluation of G03 draft genome by mapping rate of short-read against the draft genome

Items	Counts
Mapping rate	92.06%
Average sequencing depth	108.19
Coverage	99.98%
Coverage (>= 5X)	99.95%
Coverage (>= 10X)	99.92%
Coverage (>= 20X)	99.86%

Table S8 InDel and SNP statistics of G03 draft genome by mapping short-reads against the draft genome

InDel/SNP	Number	Percentage (%)	Percentage of genome (%)
All InDel	59,935	100.0000	0.0058
Heterozygous InDel	56,850	94.8528	0.0055
Homozygous InDel	3,085	5.1472	0.0003
All SNP	315,356	100.0000	0.0304
Heterozygous SNP	313,491	99.4086	0.0302
Homozygous InDel	1,865	0.5914	0.0002
All InDel & SNP	375,291	100.0000	0.0362
Heterozygous InDel & SNP	370,341	98.6810	0.0357
Homozygous InDel	4950	1.3290	0.0005

Table S9 Assembly evaluation of G03 draft genome by BLAST against BUSCOs database

Term	Gene Counts	Percentage (%)
Complete BUSCOs	1178	73.0
Complete and single-copy BUSCOs	1165	72.2
Complete and duplicated BUSCOs	13	0.8
Fragmented BUSCOs	48	3.0
Missing BUSCOs	388	24.0
Total BUSCO groups searched	1614	100.0

Table S10 Mapping statistics of the Hi-C sequencing data against contigs of scaffolds

Type	Reads number	Proportion (%)
Clean Data	758,375,902	100
Paired data	730,837,630	96.37
Mapped paired data	201,724,768	27.60
Map paired data (map Q>=5)	165,545,998	22.65

Note: Mapped paired data indicating that paired data with mate mapped to a different contig or scaffold.

Table S11 Statistics of super-scaffolds assembly of G03

Super-scaffold	Number of contigs	Length of contigs	Length of super-scaffold
Superscaffold1	17	133,088,707	133,096,707
Superscaffold2	4	105,624,284	105,625,784
Superscaffold3	6	73,574,181	73,576,681
Superscaffold4	15	69,710,716	69,717,716
Superscaffold5	3	62,505,212	62,506,212
Superscaffold6	6	52,794,264	52,796,764
Superscaffold7	7	51,198,707	51,201,707
Superscaffold8	11	50,588,079	50,593,079
Superscaffold9	13	50,292,580	50,298,580
Superscaffold10	15	50,115,551	50,122,551
Superscaffold11	14	48,633,794	48,640,294
Superscaffold12	7	45,097,437	45,100,437
Superscaffold13	15	43,668,728	43,675,728
Superscaffold14	16	43,106,541	43,114,041
Superscaffold15	8	40,288,583	40,292,083
Superscaffold16	4	36,289,572	36,291,072
Superscaffold17	20	34,175,717	34,185,217
Superscaffold18	17	32,035,679	32,043,679
Total	198	1,022,788,332	1,022,878,332

Table S12 Statistics of repetitive sequences in G03 draft genome

Predicting methods	Repeat size (bp)	% of genome
Trf	32,741,758	3.16
Repeatmasker	25,022,481	2.41
Proteinmask	124,728,081	12.03
De novo	792,927,242	76.47
Total	808,204,710	77.94

Table S13 Class and number of repeat elements in G03 draft genome by de novo analysis

Class of Repeat sequence	RepeatMasker TEs		Repeat ProteinMask TEs		De novo		Combined TEs	
	Length (bp)	Ratio	Length (bp)	Ratio	Length (bp)	Ratio	Length (bp)	Ratio
DNA	9,361,970	0.90	270,073	0.03	72,760,918	7.02	79,225,796	7.64
LINE	1,352,453	0.13	375,022	0.04	44,860,155	4.33	46,195,857	4.46
SINE	20,427	0.00	0	0.00	0	0.00	20,427	0.00
LTR	15,929,377	1.54	124,083,631	11.97	640,668,054	61.79	648,583,027	62.55
Unknown	18,405	0.00	0	0.00	115,680,636	11.16	115,697,539	11.16
Total TE	24,800,258	2.39	124,728,081	12.03	792,497,958	76.43	802,800,918	77.42

Table S14 Predicting protein-coding genes in G03 draft genome

Gene set	Number	Average gene length (bp)	Average CDS length (bp)	Average exon per gene	Average exon length (bp)	Average intron length (bp)
denovo/AUGUSTUS	22719	17802.76	1075.2	4.99	215.32	4188.57
denovo/GlimmerHMM	45875	21245.06	641.35	4.35	147.34	6145.09
homo/A.shenzhenica	22621	9211.88	870.45	3.58	242.97	3229.91
homo/D.catenatum	25284	8584.85	857.7	3.37	254.31	3256.77
homo/A.officinalis	17974	9920.56	888.32	4.1	216.59	2912.29
homo/P.equestris	19978	10204.04	955.87	4.15	230.45	2937.87
trans.orf/RNAseq	11831	17816.35	1156.03	5.77	330.67	3336.12
MAKER	17894	18384.53	1120.12	5.64	282.29	3622.35

Table S15 Statistics of non-coding RNA genes in G03 draft genome

Type		Copy	Average length (bp)	Total length (bp)	% of Genome
miRNA		25	115.20	2,880	0.0003
tRNA		1,098	73.76	80,984	0.0078
rRNA	Total	204	480.45	98,011	0.0095
	18S	88	904.75	79,618	0.0077
	28S	49	193.39	9,476	0.0009
	5.8S	28	155.64	4,358	0.0004
	5S	39	116.90	4,559	0.0004
snRNA	Total	189	108.39	20,485	0.0020
	CD-box	52	103.52	5,383	0.0005
	HACA-box	3	142.00	426	0.0000
	splicing	134	109.52	14,676	0.0014

Table S16 Annotation of predicting genes in G03 draft genome

Type		Number	Percent (%)
Total		17,894	100.00
Annotated database	InterPro	12,696	70.95
	GO	10,858	60.68
	KEGG_ALL	14,789	82.65
	KEGG_KO	6,309	35.26
	Swissprot	11,252	62.88
	TrEMBL	14,801	82.71
	NR	14,998	83.82
Annotated		15,018	83.93
Unannotated		2,876	16.07

Table S17 Summary of resequencing data of 29 accessions *GE* and their mapping ratio against reference genome

Sample Code	Sampling Name	Clean Reads Pairs Number	Clean Base (bp)	Q20 (%)	Q30 (%)	Mapped (%)	Properly mapped (%)	Singletons mapped (%)
G01	*G_viridis*_YNYL	97,491,474	14,512,112,691	98.0	93.4	77.98	70.37	0.69
G02	*G_glauca*_YNYL	74,389,908	11,089,497,749	98.1	93.5	99.36	92.27	0.21
G04	*G_viridis*_HBYCa	74,103,414	11,036,246,134	98.2	93.8	99.49	97.44	0.03
G05	*G_elata*_GZZY	89,202,496	13,315,694,135	98.8	95.7	98.80	96.70	0.03
G06	*G_viridis*_GZZY	66,988,636	9,982,484,163	98.3	94.1	98.14	96.12	0.04
G07	*G_elata*_SXNQ	78,174,292	11,652,027,724	98.2	93.6	97.61	95.61	0.04
G08	*G_viridis*_SXNQ	78,957,504	11,764,001,322	98.2	93.9	97.98	95.93	0.04
G09	*G_viridis*-HBYL	66,465,290	9,907,344,539	98.0	93.1	99.11	96.89	0.04
G10	*G_elata*_SXQL	94,021,936	14,011,389,517	97.9	93.1	98.39	95.99	0.05
G11	*G_glauca*_HBYL	108,451,008	16,169,838,168	98.1	93.4	99.00	92.05	0.23
G12	*G_elata*_HBYCb	72,397,260	10,786,808,833	98.3	94.2	97.80	95.39	0.05
G13	*G_elata*_HNNYa	99,408,080	14,822,596,212	98.2	93.6	99.53	96.91	0.04
G14	*G_elata*_SXMX	76,077,124	11,337,619,507	98.3	94.2	99.73	97.37	0.03
G15	*G_elata*_HNNYb	76,446,214	11,396,865,862	98.1	93.4	97.17	94.95	0.05
G16	*G_glauca*_AHLA	92,669,364	13,809,052,121	98.4	94.2	99.16	92.65	0.23
G17	*G_elata*_SCGY	84,519,280	12,594,241,249	98.3	94.0	96.95	94.94	0.05

G18	*G_elata*_GZTJa	86,823,100	12,940,362,844	98.0	93.1	98.48	96.18	0.05
G19	*G_glauca*_GZTJa	67,534,362	10,062,965,165	98.4	94.3	98.53	91.94	0.26
G20	*G_viridis*_GZDF	72,484,542	10,808,895,605	98.3	94.1	98.93	92.15	0.25
G21	*G_glauca*_GZDF	72,284,530	10,770,746,337	98.3	94.1	98.17	91.45	0.23
G22	*G_elata*_GZSB	80,739,584	12,037,347,129	98.3	93.9	98.26	96.15	0.04
G23	*G_viridis*_GZSB	81,880,186	12,194,047,499	98.2	93.6	96.97	94.47	0.06
G24	*G_viridis*_GZSS	65,948,476	9,823,121,323	98.2	93.7	99.93	98.24	0.02
G25	*G_glauca*_GZTJb	70,498,394	10,502,417,794	98.3	94.0	94.37	87.54	0.28
G26	*G_viridis*_GZTJb	65,076,398	9,695,045,415	98.4	94.2	99.23	92.19	0.23
G27	*G_glauca*_SCMY	101,350,738	15,012,218,860	97.0	91.2	98.36	90.41	0.24
G28	*G_glauca*_HBYLb	57,268,476	8,508,390,638	98.2	93.9	93.64	86.01	0.28
G29	*G_glauca*_GZLS	70,465,476	10,512,227,171	98.3	94.0	87.85	83.03	0.19
G30	*G_viridis*_GZLS	70,674,150	10,543,185,876	98.3	94.1	86.92	81.84	0.24

Table S18 Statistics of mapping coverage depth of 29 *GE* accessions resequencing data against reference genome

Sample Code	Sample Name	Average sequencing depth (x)	Coverage (%)	Coverage at least 4X (%)	Coverage at least 10X	Coverage at least 20X (%)
G01	*G_viridis*_YNYL	9.24	92.72	86.82	41.31	2.95
G02	*G_glauca*_YNYL	8.90	92.09	83.35	36.81	2.83
G04	*G_viridis*_HBYCa	9.71	99.16	93.15	40.54	1.40
G05	*G_elata*_GZZY	11.01	99.12	95.22	53.36	2.50
G06	*G_viridis*_GZZY	8.50	98.85	89.86	30.01	1.04
G07	*G_elata*_SXNQ	9.84	99.25	93.35	41.95	1.58
G08	*G_viridis*_SXNQ	9.87	99.17	92.74	43.02	1.99
G09	*G_viridis*-HBYL	8.14	98.96	87.14	23.74	0.88
G10	*G_elata*_SXQL	11.35	99.48	94.99	51.59	2.96
G11	*G_glauca*_HBYL	12.78	92.93	88.19	63.57	9.47
G12	*G_elata*_HBYCb	9.29	99.25	88.85	26.18	0.99
G13	*G_elata*_HNNYa	12.24	99.50	96.44	60.67	4.65
G14	*G_elata*_SXMX	9.98	99.11	91.24	35.97	1.37
G15	*G_elata*_HNNYb	9.18	99.14	91.56	36.22	1.33
G16	*G_glauca*_AHLA	11.49	92.22	86.25	53.83	6.00
G17	*G_elata*_SCGY	10.49	98.99	91.85	40.96	1.84
G18	*G_elata*_GZTJa	10.76	99.14	91.52	35.94	1.27
G19	*G_glauca*_GZTJa	8.52	91.43	79.57	29.75	2.01
G20	*G_viridis*_GZDF	9.00	91.87	82.17	33.77	2.30
G21	*G_glauca*_GZDF	9.00	91.84	82.92	35.90	2.52
G22	*G_elata*_GZSB	10.08	99.28	92.94	38.53	1.25
G23	*G_viridis*_GZSB	10.18	99.09	92.42	38.65	1.37
G24	*G_viridis*_GZSS	8.70	98.95	89.96	34.72	1.36
G25	*G_glauca*_GZTJb	8.63	91.65	80.96	32.26	2.52
G26	*G_viridis*_GZTJb	8.27	91.65	80.43	28.71	2.13
G27	*G_glauca*_SCMY	12.38	92.86	87.68	61.65	9.13
G28	*G_glauca*_HBYLb	6.51	91.29	74.93	16.18	1.29
G29	*G_glauca*_GZLS	7.48	90.14	75.21	23.55	1.61
G30	*G_viridis*_GZLS	7.45	90.20	74.67	20.23	1.41

Table S19 Genetic diversity and differentiation of *GEV* and non-*GEV* subpopulations

Parameters	Population name	
	G. elata Bl.f.viridis	Non-*G. elata Bl.f.viridis*
Pi	2.915×10^{-4}	3.175×10^{-4}
Tajiam`D	-0.37126	-0.14956
F_{ST}	0.0033	

Table S20 Summary of genomic bin (1 bin=100K) with high differentiation

Methods and criterions	Bin Number	Gene Number
F_{ST} (Top 1%)	167	291
F_{ST} (Top 5%)	498	979
dXY (Top 1%)	185	305
dXY (Top 5%)	497	964
F_{ST} & dXY (Top 1%)	154	267
F_{ST} & dXY (Top 5%)	448	863
Pi ratio of Pop1/Pop2 (Top 1%)	256	430
Pi ratio of Pop1/Pop2 (Top 5%)	432	740
Pi ratio of Pop2/Pop1 (Top 1%)	98	240
Pi ratio of Pop2/Pop1 (Top 5%)	173	442
F_{ST} & Pi ratio of Pop1/Pop2 (Top 1%)	79	137
F_{ST} & Pi ratio of Pop1/Pop2 (Top 5%)	219	360
F_{ST} & ratio of Pi Pop2/Pop1 (Top 1%)	5	5
F_{ST} & Pi ratio of Pop2/Pop1 (Top 5%)	17	20

Note: Pop 1: Population including samples of all *GEG* samples or *GEV* samples grow scattered in *GEG;* Pop 2: Population including samples of all *GEE* samples or *GEV* grow scattered in *GEE*.
